# Supplementary material for: Multiplexed single-molecule analysis of barcoded protein variants
Source: Nat Commun. 2026 Oct 12;17:11560. doi: 10.1038/s41467-026-78581-y (PMC13748599; doi:10.1038/s41467-026-78581-y)
Supplement: Supplementary file 1 [file 41467_2026_78581_MOESM1_ESM.pdf]

Supplementary Information

Multiplexed single-molecule analysis of barcoded protein variants

Stefan H. Mueller[1], David M. Grimson[1], Holden Paz[1], Aidan C. Fitch[1], Haibo Yu[1,2], Marco Ribezzi-Crivellari[3], Andrew D. Griffiths[3], Antoine M. van Oijen[1], Lisanne M. Spenkelink[1]

[1]Molecular Horizons and School of Science, University of Wollongong, Wollongong, NSW 2522, Australia.

[2]ARC Centre of Excellence in Quantum Biotechnology, University of Wollongong, Wollongong, NSW 2522, Australia

[3]Laboratoire de Biochimie, Chimie Biologie et Innovation, ESPCI Paris, Université PSL, Paris, France.

*Correspondence to: lisanne@uow.edu.au.

This file contains Supplementary Figs. 1–7 and Supplementary Tables 1–4.

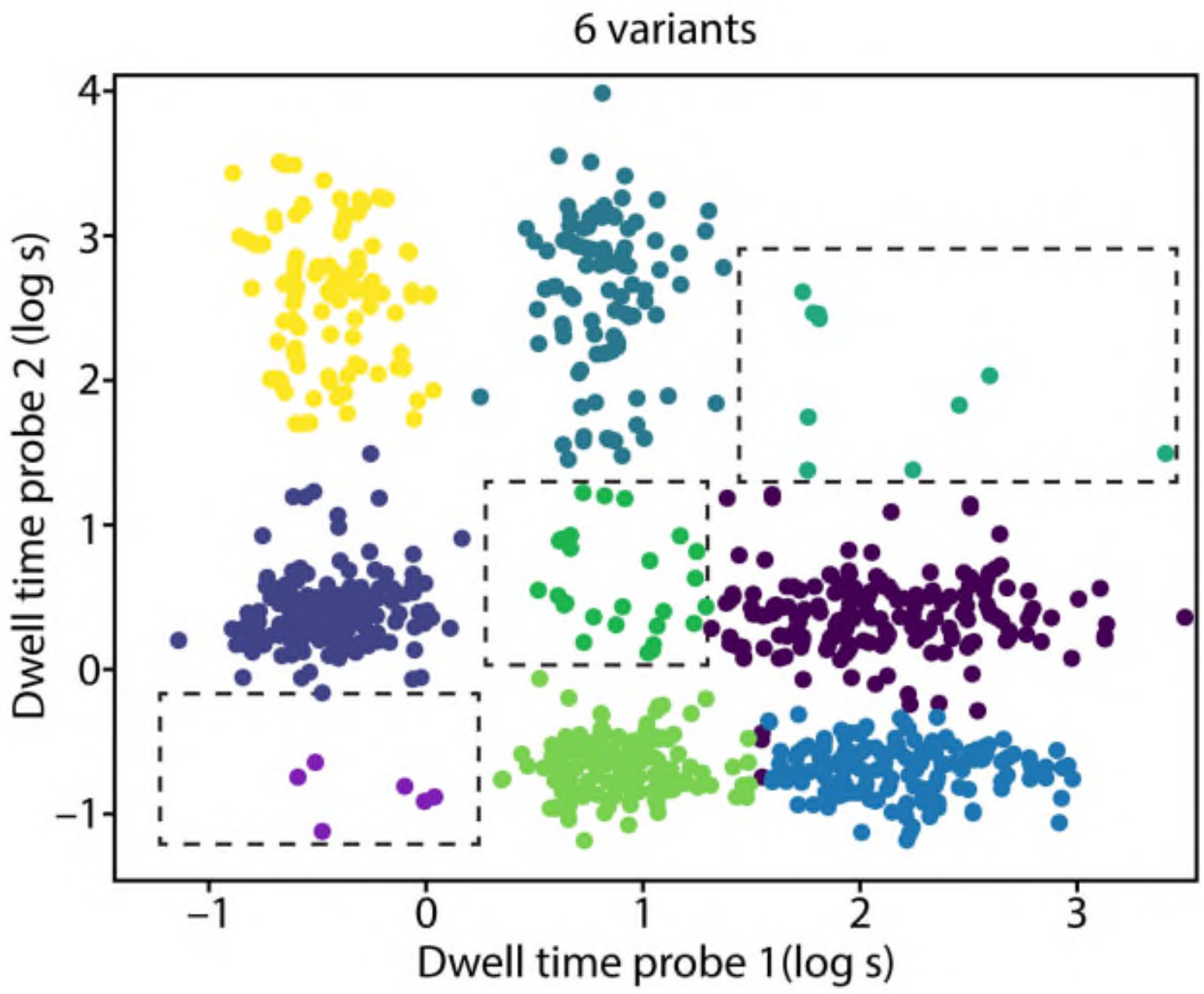

Supplementary Fig. 1. Quantification of accuracy. We measured classification accuracy using six two-index barcodes, instead of nine (Fig. 2), excluding barcodes with both indices of the same length (7nt–7nt, 8nt–8nt, and 9nt–9nt; dashed boxes). Each dot in the graph represents a single two-index barcode. Each colour represents a cluster of identical barcodes. Clusters are defined to be 1.5 standard deviations around the cluster means (Supplementary table 2). Any dot appearing in the dashed boxes represents a false call. Based on this, we observed an accuracy exceeding 95% (*e.g.* 5% falls calls). Source data are provided as a Source Data file.

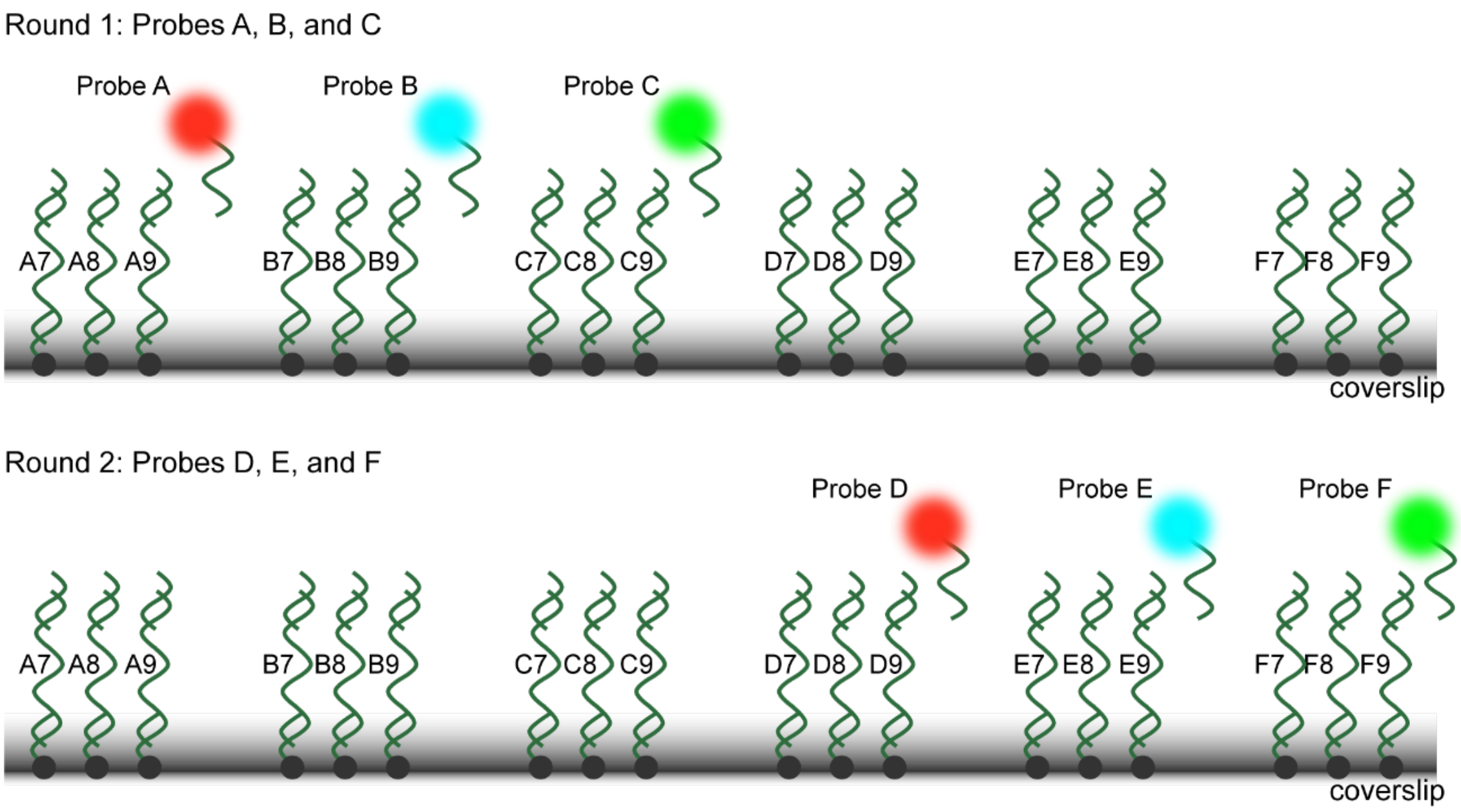

Supplementary Fig. 2. Measurement of 18 unique barcodes using six orthogonal probes. We generated a set of 18 indices, by combining six orthogonal probes (A–F), each capable of binding three distinct single-index DNA barcodes with 7-nt, 8-nt, or 9-nt complementarity to the probe. All 18 barcodes are immobilised on the microscope coverslip simultaneously. Probes A–C are imaged in the first round (top panel), and probes D–F in the second round (bottom panel). By using three spectrally distinct fluorophores (two probes per

colour) and resolving binding kinetics for each probe, we generate 6 × 3 = 18 unique indices. Single indices are shown for simplicity; the same workflow applies to multi-index barcodes.

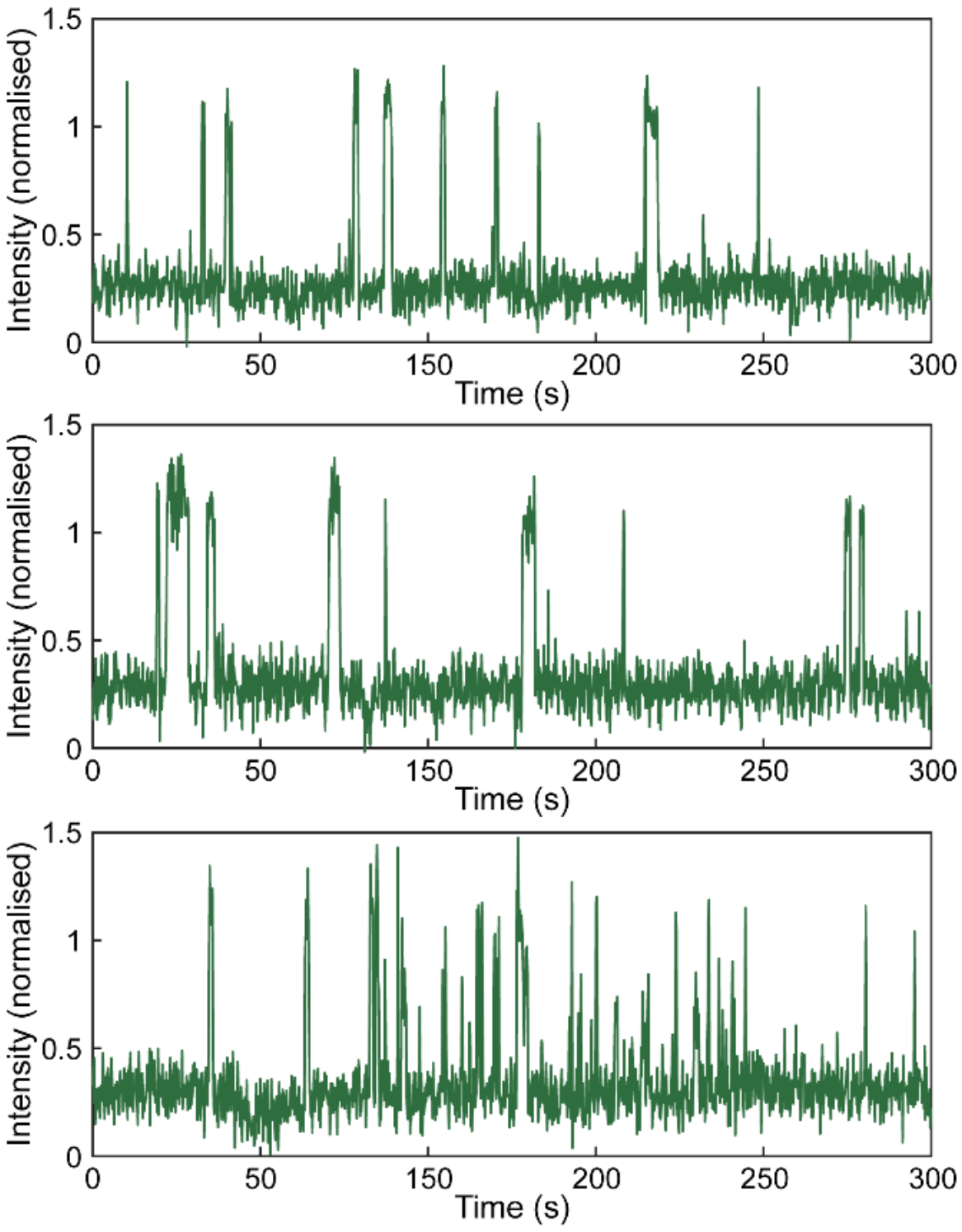

Supplementary Fig. 3. Representative trajectories showing peptide binding to ClpS. Three representative trajectories showing the normalised fluorescence intensity of peptides binding to ClpS. Source data are provided as a Source Data file.

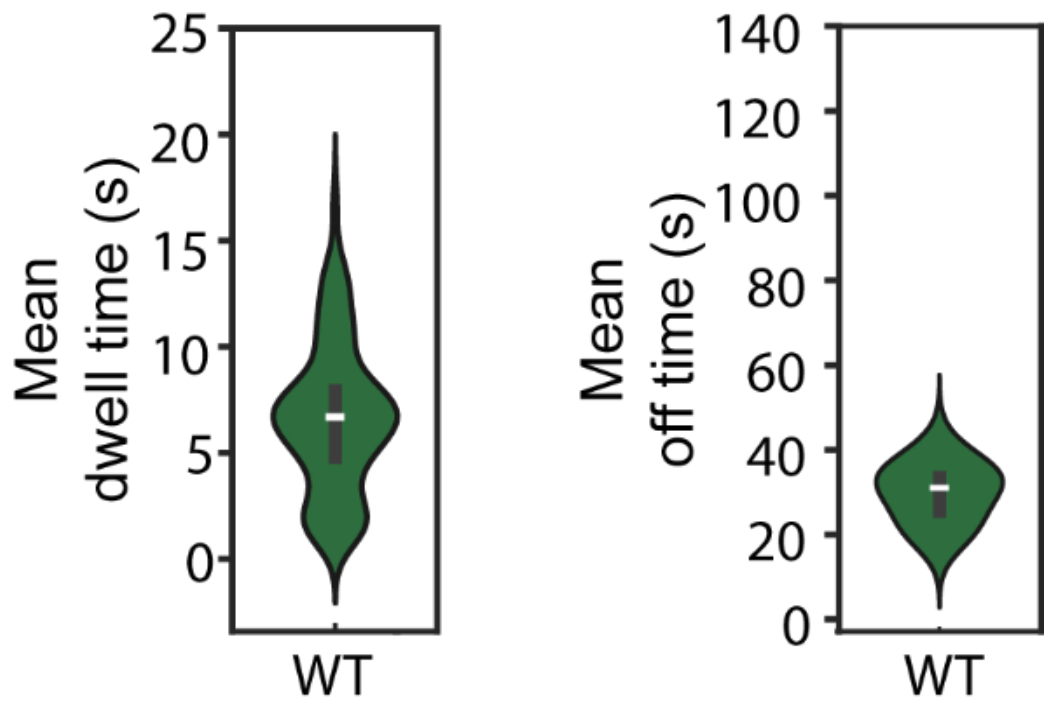

Supplementary Fig. 4. Measurement of wild-type ClpS in isolation. A measurement of the peptide binding kinetics in an experiment that contained only wild-type ClpS, reproduce the distributions of mean dwell times (left) and mean off times obtained in Fig. 3c. Source data are provided as a Source Data file.

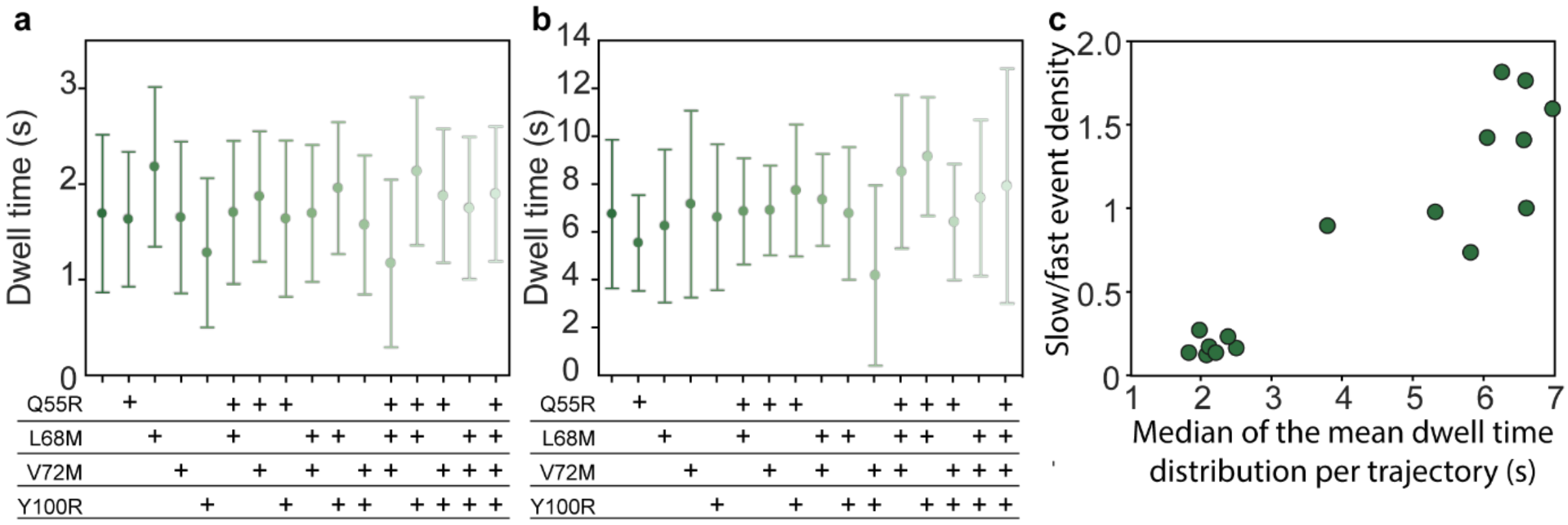

Supplementary Fig. 5. Quantification of the bimodal dwell distributions. The violin plots for the dwell time distributions shown in Fig. 3e were fit with the sum of two Gaussians to calculate **a**, the mean dwell time of the fast population for each variant (~1.5 s for all variants), and **b**, the mean dwell time of the slow population for each variant (~7s for all variants). Error bars represent the standard deviation. **c**, the ratio of the densities for the slow and fast populations *vs* the median of the mean dwell time distributions per trajectory, shows that the dwell time is largely governed by the ratio of the two kinetic states. Source data are provided as a Source Data file.

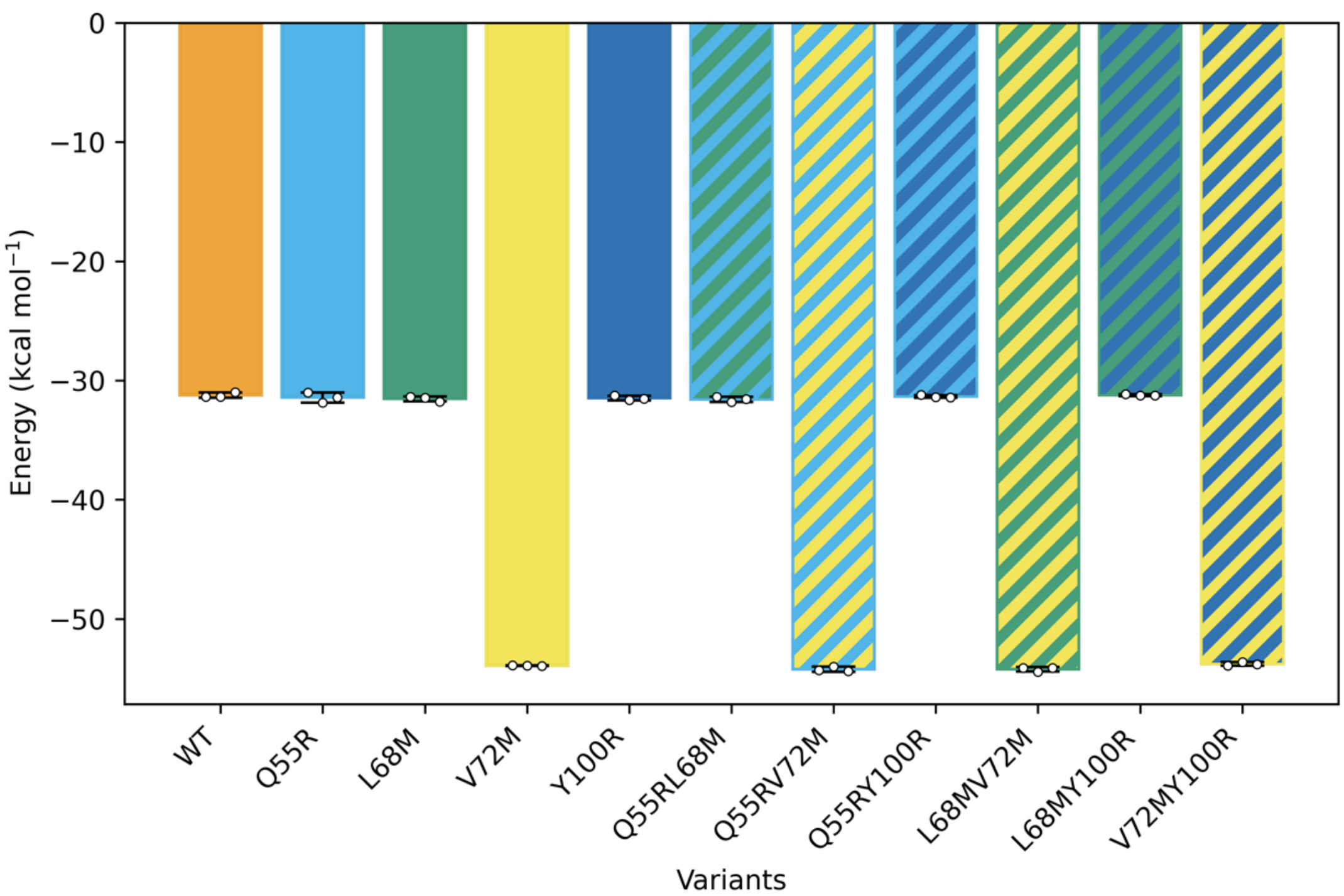

Supplementary Fig. 6. Electrostatic interaction energies of the terminal leucine residue with ClpS Val/Met72. This figure depicts the component of the interaction energy between the ClpS residue 72 (which can be either Val or Met depending on the variant) and the N-terminal leucine residue of the peptide. Each data point (the circles on the bars) is the average of this component of the interaction energy across the 50,000 trajectory frames of a single replicate calculated with the enedecomp module of CPPTRAJ. The bars represent the means of the triplicate data, and the error bars represent the standard deviations. There is a significant decrease in energy with mutation from the wild-type valine to methionine. This decrease in energy is not affected by additional mutations. Source data are provided as a Source Data file.

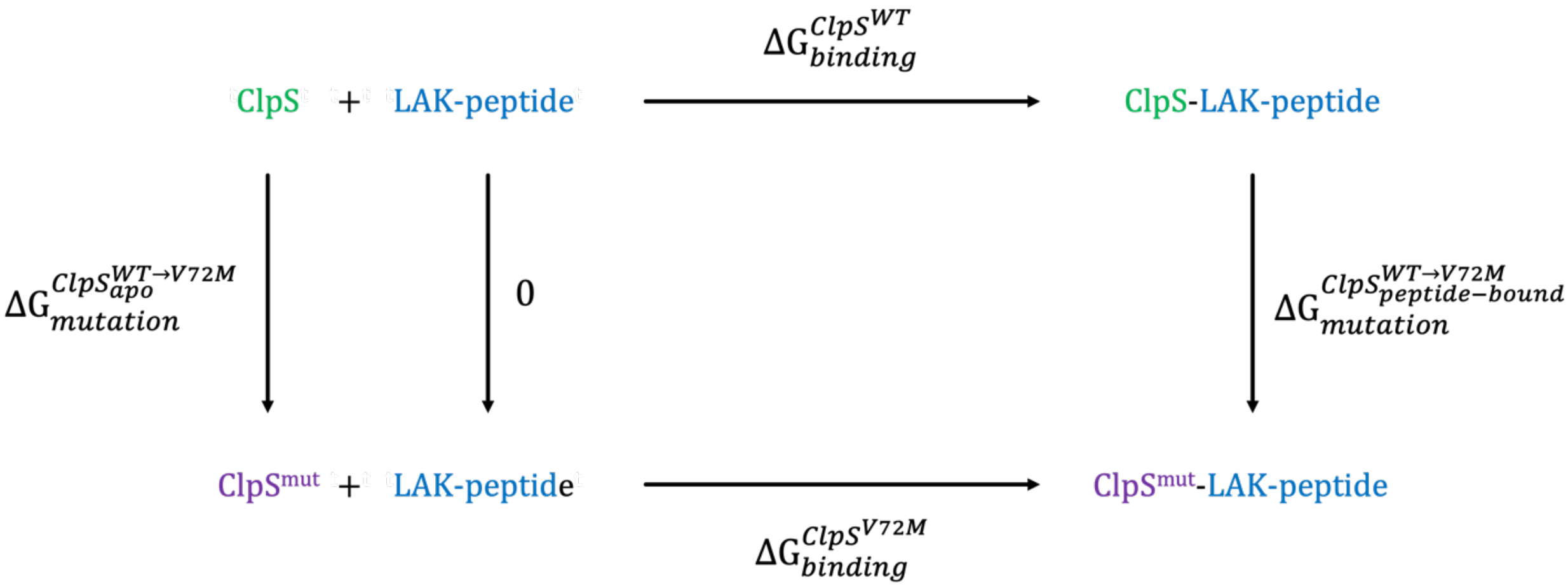

Supplementary Fig. 7. Thermodynamic cycle used in alchemical thermodynamic integration simulations. Wild-type ClpS is transformed to the variant ClpS^{V72M} in apo and in the peptide-bound state. The simulations involve the vertical alchemical transformations.

Supplementary Table 1. Index and probe sequences

**Barcode A**	
7-nt index	/5Biosg/TTTTTTTTTTTTTTT TGT TCC T
8-nt index	/5Biosg/TTTTTTTTTTTTTTT TGT TCC TC
9-nt index	/5Biosg/TTTTTTTTTTTTTTT TGT TCC TCT
Probe A	/5Alex647N/CGCAGAGGAACA
**Barcode B**	
7-nt index	/5Biosg/TTTTTTTTTTTTTTT CTC TCC C
8-nt index	/5Biosg/TTTTTTTTTTTTTTT CTC TCC CT
9-nt index	/5Biosg/TTTTTTTTTTTTTTT CTC TCC CTC
Probe B	/5Alex488N/TT GAGGGAGAG
**Barcode C**	
7-nt index	/5Biosg/TTTTTTTTTTTTTTT GCT TGT C
8-nt index	/5Biosg/TTTTTTTTTTTTTTT GCT TGT CT
9-nt index	/5Biosg/TTTTTTTTTTTTTTT GCT TGT CTC
Probe C	/5Alex594N/TTGAG ACA AGC
**Barcode D**	
7-nt index	/5Biosg/TTTTTTTTTTTTTTT TGT GGT G
8-nt index	/5Biosg/TTTTTTTTTTTTTTT TGT GGT GT
9-nt index	/5Biosg/TTTTTTTTTTTTTTT TGT GGT GTT
Probe D	/Alex647N/TTAAC ACC ACA
**Barcode E**	

7-nt index	/5Biosg/TTTTTTTTTTTTTTT GCT TCT T
8-nt index	/5Biosg/TTTTTTTTTTTTTTT GCT TCT TC
9-nt index	/5Biosg/TTTTTTTTTTTTTTT GCT TCT TCC
Probe E	/5Alex488N/TTGGA AGA AGCTT
**Barcode F**	
7-nt index	/5Biosg/TTTTTTTTTTTTTTT GGT TCT C
8-nt index	/5Biosg/TTTTTTTTTTTTTTT GGT TCT CT
9-nt index	/5Biosg/TTTTTTTTTTTTTTT GGT TCT CTG
Probe F	/5Alex594N/TTCAG AGA ACC

Supplementary Table 2. Dwell times (s) for all indices.

	Index length (nt)			
**Barcode**		**7**	**8**	**9**
	**A**	0.34 ± 0.05	2.39 ± 0.02	8.01 ± 0.44
	**B**	0.46 ± 0.05	1.44 ± 0.08	20.43 ± 1.39
	**C**	1.40 ± 0.30	7.70 ± 2.20	38.10 ± 5.90
	**D**	0.58 ± 0.03	3.01 ± 0.13	20.37 ± 0.28
	**E**	2.70 ± 0.20	9.70 ± 0.08	74.73 ± 17.08
	**F**	3.00 ± 1.50	9.00 ± 2.70	23.89 ± 2.29

Supplementary Table 3: PCR primers for SNAP–ClpS constructs

Barcode_FW	GATACCAGGCGTTTCCC
Barcode_RV	GCCAGTGCCAAGCTTC
RecA_FW	ACCCGGCATGACAGGAG
RecA_RV	GTGGCGGCCGCTCTA

Supplementary Table 4: SNAP–ClpS constructs

WT	ACCCGGCATGACAGGAGGATCGAGGCCTCTTAATACGACTCACTATAGGGGAATTG<br>TGAGCGGATAACAATTCCCCTCTAGAAATAATTTTGTTTAACTTTAAGAAGGAGATATA<br>CATATGGACAAAGACTGCGAAATGAAACGTACCACCCTGGACTCTCCGCTGGGTAA<br>ACTGGAACTGTCTGGTTGCGAACAGGGTCTGCACGAAATCATCTTCCTGGGTAAAG<br>GTACCTCTGCGGCGGACGCGGTTGAAGTTCCCGCACCGGCTGCGGTTCTGGGTG<br>GTCCGGAACCGCTGATGCAGGCGACCGCGTGGCTGAACGCGTACTTCCACCAGC<br>CGGAAGCGATCGAAGAATTCCCGGTTCCGGCGCTGCACCACCCGGTTTTCCAGCA<br>GGAATCTTTCACCCGTCAGGTCTTGTGGAAACTGCTGAAAGTTGTTAAATTCGGTGA<br>AGTTATCTCTTACTCTCACCTGGCGGCGCTGGCGGGTAACCCGGCAGCGACCGCG<br>GCAGTTAAAACCGCGCTGTCTGGTAACCCGGTTCCGATCCTGATCCCGTGCCACCG<br>TGTTGTTAACATCAACGGTACCGTTGGTGGTTACGAAGGTGGTCTGGCGGTTAAAG<br>AATGGCTGCTGGCGCACGAAGGTCACCGTCTGGGTAAACCGGGTCTGGGTGGTAC<br>CGGCGGTCTGGTTCCACGTGGCTCCGGAGGCTCTGCGGGTTCTGCAGCTGGTTCT<br>GAATTCATGCCAACAGCAGCTTCTGCAACGGAATCAGCAATTGAAGACACACCAGC<br>TCCTGCCCGCCCTGAGGTTGATGGCCGCACAAAACCAAAGCGCCAACCACGCTAT<br>CATGTTGTACTCTGGGACGACGACGATCATACATATCAATACGTTGTGGTTATGCTCC<br>AAAGTTTGTTCGGCCATCCACCCGAACGCGGTTACCGCCTCGCAAAAGAGGTTGA<br>CACTCAAGGACGCGTGATTGTATTGACAACGACTCGCGAACATGCCGAGCTTAAGC<br>GCGACCAGATCCATGCATTCGGGTATGATCGCCTTCTTGCACGCAGTAAAGGTTCG<br>ATGAAGGCGTCCATCGAGGCCGAAGAGGGCGGTGGCAGCGGTGGCGGTAGCGGC<br>GGTGGCAGCGGCGGCCTGAACGATATTTTTGAAGCGCAGAAAATTGAATGGCATGA<br>AGATTATAAAGATCATGATGGTGATTATAAAGATCACGATATCGACTACAAAGACGATG<br>ACGACAAGTGA
Q55R	ACCCGGCATGACAGGAGGATCGAGGCCTCTTAATACGACTCACTATAGGGGAATTG<br>TGAGCGGATAACAATTCCCCTCTAGAAATAATTTTGTTTAACTTTAAGAAGGAGATATA<br>CATATGGACAAAGACTGCGAAATGAAACGTACCACCCTGGACTCTCCGCTGGGTAA<br>ACTGGAACTGTCTGGTTGCGAACAGGGTCTGCACGAAATCATCTTCCTGGGTAAAG<br>GTACCTCTGCGGCGGACGCGGTTGAAGTTCCCGCACCGGCTGCGGTTCTGGGTG<br>GTCCGGAACCGCTGATGCAGGCGACCGCGTGGCTGAACGCGTACTTCCACCAGC

	CGGAAGCGATCGAAGAATTCCCGGTTCCGGCGCTGCACCACCCGGTTTTCCAGCA<br>GGAATCTTTCACCCGTCAGGTCTTGTGGAAACTGCTGAAAGTTGTTAAATTCGGTGA<br>AGTTATCTCTTACTCTCACCTGGCGGCGCTGGCGGGTAACCCGGCAGCGACCGCG<br>GCAGTTAAAACCGCGCTGTCTGGTAACCCGGTTCCGATCCTGATCCCGTGCCACCG<br>TGTTGTTAACATCAACGGTACCGTTGGTGGTTACGAAGGTGGTCTGGCGGTTAAAG<br>AATGGCTGCTGGCGCACGAAGGTCACCGTCTGGGTAAACCGGGTCTGGGTGGTAC<br>CGGCGGTCTGGTTCCACGTGGCTCCGGAGGCTCTGCGGGTTCTGCAGCTGGTTCT<br>GAATTCATGCCAACAGCAGCTTCTGCAACGGAATCAGCAATTGAAGACACACCAGC<br>TCCTGCCCGCCCTGAGGTTGATGGCCGCACAAAACCAAAGCGCCAACCACGCTAT<br>CATGTTGTACTCTGGGACGACGACGATCATACATATCAATACGTTGTGGTTATGCTCC<br>GAAGTTTGTTCGGCCATCCACCCGAACGCGGTTACCGCCTCGCAAAAGAGGTTGA<br>CACTCAAGGACGCGTGATTGTATTGACAACGACTCGCGAACATGCCGAGCTTAAGC<br>GCGACCAGATCCATGCATTCGGGTATGATCGCCTTCTTGCACGCAGTAAAGGTTCG<br>ATGAAGGCGTCCATCGAGGCCGAAGAGGGCGGTGGCAGCGGTGGCGGTAGCGGC<br>GGTGGCAGCGGCGGCCTGAACGATATTTTTGAAGCGCAGAAAATTGAATGGCATGA<br>AGATTATAAAGATCATGATGGTGATTATAAAGATCACGATATCGACTACAAAGACGATG<br>ACGACAAGTGA
L68M	ACCCGGCATGACAGGAGGATCGAGGCCTCTTAATACGACTCACTATAGGGGAATTG<br>TGAGCGGATAACAATTCCCCTCTAGAAATAATTTTGTTTAACTTTAAGAAGGAGATATA<br>CATATGGACAAAGACTGCGAAATGAAACGTACCACCCTGGACTCTCCGCTGGGTAA<br>ACTGGAACTGTCTGGTTGCGAACAGGGTCTGCACGAAATCATCTTCCTGGGTAAAG<br>GTACCTCTGCGGCGGACGCGGTTGAAGTTCCCGCACCGGCTGCGGTTCTGGGTG<br>GTCCGGAACCGCTGATGCAGGCGACCGCGTGGCTGAACGCGTACTTCCACCAGC<br>CGGAAGCGATCGAAGAATTCCCGGTTCCGGCGCTGCACCACCCGGTTTTCCAGCA<br>GGAATCTTTCACCCGTCAGGTCTTGTGGAAACTGCTGAAAGTTGTTAAATTCGGTGA<br>AGTTATCTCTTACTCTCACCTGGCGGCGCTGGCGGGTAACCCGGCAGCGACCGCG<br>GCAGTTAAAACCGCGCTGTCTGGTAACCCGGTTCCGATCCTGATCCCGTGCCACCG<br>TGTTGTTAACATCAACGGTACCGTTGGTGGTTACGAAGGTGGTCTGGCGGTTAAAG<br>AATGGCTGCTGGCGCACGAAGGTCACCGTCTGGGTAAACCGGGTCTGGGTGGTAC<br>CGGCGGTCTGGTTCCACGTGGCTCCGGAGGCTCTGCGGGTTCTGCAGCTGGTTCT<br>GAATTCATGCCAACAGCAGCTTCTGCAACGGAATCAGCAATTGAAGACACACCAGC<br>TCCTGCCCGCCCTGAGGTTGATGGCCGCACAAAACCAAAGCGCCAACCACGCTAT<br>CATGTTGTACTCTGGGACGACGACGATCATACATATCAATACGTTGTGGTTATGCTCC<br>AAAGTTTGTTCGGCCATCCACCCGAACGCGGTTACCGCATGGCAAAAGAGGTTGAC<br>ACTCAAGGACGCGTGATTGTATTGACAACGACTCGCGAACATGCCGAGCTTAAGCG<br>CGACCAGATCCATGCATTCGGGTATGATCGCCTTCTTGCACGCAGTAAAGGTTCGAT<br>GAAGGCGTCCATCGAGGCCGAAGAGGGCGGTGGCAGCGGTGGCGGTAGCGGCG<br>GTGGCAGCGGCGGCCTGAACGATATTTTTGAAGCGCAGAAAATTGAATGGCATGAA<br>GATTATAAAGATCATGATGGTGATTATAAAGATCACGATATCGACTACAAAGACGATGA<br>CGACAAGTGA
V72M	ACCCGGCATGACAGGAGGATCGAGGCCTCTTAATACGACTCACTATAGGGGAATTG<br>TGAGCGGATAACAATTCCCCTCTAGAAATAATTTTGTTTAACTTTAAGAAGGAGATATA<br>CATATGGACAAAGACTGCGAAATGAAACGTACCACCCTGGACTCTCCGCTGGGTAA<br>ACTGGAACTGTCTGGTTGCGAACAGGGTCTGCACGAAATCATCTTCCTGGGTAAAG<br>GTACCTCTGCGGCGGACGCGGTTGAAGTTCCCGCACCGGCTGCGGTTCTGGGTG<br>GTCCGGAACCGCTGATGCAGGCGACCGCGTGGCTGAACGCGTACTTCCACCAGC<br>CGGAAGCGATCGAAGAATTCCCGGTTCCGGCGCTGCACCACCCGGTTTTCCAGCA<br>GGAATCTTTCACCCGTCAGGTCTTGTGGAAACTGCTGAAAGTTGTTAAATTCGGTGA<br>AGTTATCTCTTACTCTCACCTGGCGGCGCTGGCGGGTAACCCGGCAGCGACCGCG<br>GCAGTTAAAACCGCGCTGTCTGGTAACCCGGTTCCGATCCTGATCCCGTGCCACCG<br>TGTTGTTAACATCAACGGTACCGTTGGTGGTTACGAAGGTGGTCTGGCGGTTAAAG<br>AATGGCTGCTGGCGCACGAAGGTCACCGTCTGGGTAAACCGGGTCTGGGTGGTAC<br>CGGCGGTCTGGTTCCACGTGGCTCCGGAGGCTCTGCGGGTTCTGCAGCTGGTTCT<br>GAATTCATGCCAACAGCAGCTTCTGCAACGGAATCAGCAATTGAAGACACACCAGC<br>TCCTGCCCGCCCTGAGGTTGATGGCCGCACAAAACCAAAGCGCCAACCACGCTAT<br>CATGTTGTACTCTGGGACGACGACGATCATACATATCAATACGTTGTGGTTATGCTCC<br>AAAGTTTGTTCGGCCATCCACCCGAACGCGGTTACCGCCTCGCAAAAGAGATGGAC<br>ACTCAAGGACGCGTGATTGTATTGACAACGACTCGCGAACATGCCGAGCTTAAGCG<br>CGACCAGATCCATGCATTCGGGTATGATCGCCTTCTTGCACGCAGTAAAGGTTCGAT

	GAAGGCGTCCATCGAGGCCGAAGAGGGCGGTGGCAGCGGTGGCGGTAGCGGCG<br>GTGGCAGCGGCGGCCTGAACGATATTTTTGAAGCGCAGAAAATTGAATGGCATGAA<br>GATTATAAAGATCATGATGGTGATTATAAAGATCACGATATCGACTACAAAGACGATGA<br>CGACAAGTGA
Y100R	ACCCGGCATGACAGGAGGATCGAGGCCTCTTAATACGACTCACTATAGGGGAATTG<br>TGAGCGGATAACAATTCCCCTCTAGAAATAATTTTGTTTAACTTTAAGAAGGAGATATA<br>CATATGGACAAAGACTGCGAAATGAAACGTACCACCCTGGACTCTCCGCTGGGTAA<br>ACTGGAACTGTCTGGTTGCGAACAGGGTCTGCACGAAATCATCTTCCTGGGTAAAG<br>GTACCTCTGCGGCGGACGCGGTTGAAGTTCCCGCACCGGCTGCGGTTCTGGGTG<br>GTCCGGAACCGCTGATGCAGGCGACCGCGTGGCTGAACGCGTACTTCCACCAGC<br>CGGAAGCGATCGAAGAATTCCCGGTTCCGGCGCTGCACCACCCGGTTTTCCAGCA<br>GGAATCTTTCACCCGTCAGGTCTTGTGGAAACTGCTGAAAGTTGTTAAATTCGGTGA<br>AGTTATCTCTTACTCTCACCTGGCGGCGCTGGCGGGTAACCCGGCAGCGACCGCG<br>GCAGTTAAAACCGCGCTGTCTGGTAACCCGGTTCCGATCCTGATCCCGTGCCACCG<br>TGTTGTTAACATCAACGGTACCGTTGGTGGTTACGAAGGTGGTCTGGCGGTTAAAG<br>AATGGCTGCTGGCGCACGAAGGTCACCGTCTGGGTAAACCGGGTCTGGGTGGTAC<br>CGGCGGTCTGGTTCCACGTGGCTCCGGAGGCTCTGCGGGTTCTGCAGCTGGTTCT<br>GAATTCATGCCAACAGCAGCTTCTGCAACGGAATCAGCAATTGAAGACACACCAGC<br>TCCTGCCCGCCCTGAGGTTGATGGCCGCACAAAACCAAAGCGCCAACCACGCTAT<br>CATGTTGTACTCTGGGACGACGACGATCATACATATCAATACGTTGTGGTTATGCTCC<br>AAAGTTTGTTCGGCCATCCACCCGAACGCGGTTACCGCCTCGCAAAAGAGGTTGA<br>CACTCAAGGACGCGTGATTGTATTGACAACGACTCGCGAACATGCCGAGCTTAAGC<br>GCGACCAGATCCATGCATTCGGGCGAGATCGCCTTCTTGCACGCAGTAAAGGTTCG<br>ATGAAGGCGTCCATCGAGGCCGAAGAGGGCGGTGGCAGCGGTGGCGGTAGCGGC<br>GGTGGCAGCGGCGGCCTGAACGATATTTTTGAAGCGCAGAAAATTGAATGGCATGA<br>AGATTATAAAGATCATGATGGTGATTATAAAGATCACGATATCGACTACAAAGACGATG<br>ACGACAAGTGA
Q55R,<br>L68M	ACCCGGCATGACAGGAGGATCGAGGCCTCTTAATACGACTCACTATAGGGGAATTG<br>TGAGCGGATAACAATTCCCCTCTAGAAATAATTTTGTTTAACTTTAAGAAGGAGATATA<br>CATATGGACAAAGACTGCGAAATGAAACGTACCACCCTGGACTCTCCGCTGGGTAA<br>ACTGGAACTGTCTGGTTGCGAACAGGGTCTGCACGAAATCATCTTCCTGGGTAAAG<br>GTACCTCTGCGGCGGACGCGGTTGAAGTTCCCGCACCGGCTGCGGTTCTGGGTG<br>GTCCGGAACCGCTGATGCAGGCGACCGCGTGGCTGAACGCGTACTTCCACCAGC<br>CGGAAGCGATCGAAGAATTCCCGGTTCCGGCGCTGCACCACCCGGTTTTCCAGCA<br>GGAATCTTTCACCCGTCAGGTCTTGTGGAAACTGCTGAAAGTTGTTAAATTCGGTGA<br>AGTTATCTCTTACTCTCACCTGGCGGCGCTGGCGGGTAACCCGGCAGCGACCGCG<br>GCAGTTAAAACCGCGCTGTCTGGTAACCCGGTTCCGATCCTGATCCCGTGCCACCG<br>TGTTGTTAACATCAACGGTACCGTTGGTGGTTACGAAGGTGGTCTGGCGGTTAAAG<br>AATGGCTGCTGGCGCACGAAGGTCACCGTCTGGGTAAACCGGGTCTGGGTGGTAC<br>CGGCGGTCTGGTTCCACGTGGCTCCGGAGGCTCTGCGGGTTCTGCAGCTGGTTCT<br>GAATTCATGCCAACAGCAGCTTCTGCAACGGAATCAGCAATTGAAGACACACCAGC<br>TCCTGCCCGCCCTGAGGTTGATGGCCGCACAAAACCAAAGCGCCAACCACGCTAT<br>CATGTTGTACTCTGGGACGACGACGATCATACATATCAATACGTTGTGGTTATGCTCC<br>GAAGTTTGTTCGGCCATCCACCCGAACGCGGTTACCGCATGGCAAAAGAGGTTGA<br>CACTCAAGGACGCGTGATTGTATTGACAACGACTCGCGAACATGCCGAGCTTAAGC<br>GCGACCAGATCCATGCATTCGGGTATGATCGCCTTCTTGCACGCAGTAAAGGTTCG<br>ATGAAGGCGTCCATCGAGGCCGAAGAGGGCGGTGGCAGCGGTGGCGGTAGCGGC<br>GGTGGCAGCGGCGGCCTGAACGATATTTTTGAAGCGCAGAAAATTGAATGGCATGA<br>AGATTATAAAGATCATGATGGTGATTATAAAGATCACGATATCGACTACAAAGACGATG<br>ACGACAAGTGA
Q55R,<br>V72M	ACCCGGCATGACAGGAGGATCGAGGCCTCTTAATACGACTCACTATAGGGGAATTG<br>TGAGCGGATAACAATTCCCCTCTAGAAATAATTTTGTTTAACTTTAAGAAGGAGATATA<br>CATATGGACAAAGACTGCGAAATGAAACGTACCACCCTGGACTCTCCGCTGGGTAA<br>ACTGGAACTGTCTGGTTGCGAACAGGGTCTGCACGAAATCATCTTCCTGGGTAAAG<br>GTACCTCTGCGGCGGACGCGGTTGAAGTTCCCGCACCGGCTGCGGTTCTGGGTG<br>GTCCGGAACCGCTGATGCAGGCGACCGCGTGGCTGAACGCGTACTTCCACCAGC<br>CGGAAGCGATCGAAGAATTCCCGGTTCCGGCGCTGCACCACCCGGTTTTCCAGCA<br>GGAATCTTTCACCCGTCAGGTCTTGTGGAAACTGCTGAAAGTTGTTAAATTCGGTGA

	AGTTATCTCTTACTCTCACCTGGCGGCGCTGGCGGGTAACCCGGCAGCGACCGCG<br>GCAGTTAAAACCGCGCTGTCTGGTAACCCGGTTCCGATCCTGATCCCGTGCCACCG<br>TGTTGTTAACATCAACGGTACCGTTGGTGGTTACGAAGGTGGTCTGGCGGTTAAAG<br>AATGGCTGCTGGCGCACGAAGGTCACCGTCTGGGTAAACCGGGTCTGGGTGGTAC<br>CGGCGGTCTGGTTCCACGTGGCTCCGGAGGCTCTGCGGGTTCTGCAGCTGGTTCT<br>GAATTCATGCCAACAGCAGCTTCTGCAACGGAATCAGCAATTGAAGACACACCAGC<br>TCCTGCCCGCCCTGAGGTTGATGGCCGCACAAAACCAAAGCGCCAACCACGCTAT<br>CATGTTGTACTCTGGGACGACGACGATCATACATATCAATACGTTGTGGTTATGCTCC<br>GAAGTTTGTTCGGCCATCCACCCGAACGCGGTTACCGCCTCGCAAAAGAGATGGA<br>CACTCAAGGACGCGTGATTGTATTGACAACGACTCGCGAACATGCCGAGCTTAAGC<br>GCGACCAGATCCATGCATTCGGGTATGATCGCCTTCTTGCACGCAGTAAAGGTTCG<br>ATGAAGGCGTCCATCGAGGCCGAAGAGGGCGGTGGCAGCGGTGGCGGTAGCGGC<br>GGTGGCAGCGGCGGCCTGAACGATATTTTTGAAGCGCAGAAAATTGAATGGCATGA<br>AGATTATAAAGATCATGATGGTGATTATAAAGATCACGATATCGACTACAAAGACGATG<br>ACGACAAGTGA
Q55R,<br>Y100R	ACCCGGCATGACAGGAGGATCGAGGCCTCTTAATACGACTCACTATAGGGGAATTG<br>TGAGCGGATAACAATTCCCCTCTAGAAATAATTTTGTTTAACTTTAAGAAGGAGATATA<br>CATATGGACAAAGACTGCGAAATGAAACGTACCACCCTGGACTCTCCGCTGGGTAA<br>ACTGGAACTGTCTGGTTGCGAACAGGGTCTGCACGAAATCATCTTCCTGGGTAAAG<br>GTACCTCTGCGGCGGACGCGGTTGAAGTTCCCGCACCGGCTGCGGTTCTGGGTG<br>GTCCGGAACCGCTGATGCAGGCGACCGCGTGGCTGAACGCGTACTTCCACCAGC<br>CGGAAGCGATCGAAGAATTCCCGGTTCCGGCGCTGCACCACCCGGTTTTCCAGCA<br>GGAATCTTTCACCCGTCAGGTCTTGTGGAAACTGCTGAAAGTTGTTAAATTCGGTGA<br>AGTTATCTCTTACTCTCACCTGGCGGCGCTGGCGGGTAACCCGGCAGCGACCGCG<br>GCAGTTAAAACCGCGCTGTCTGGTAACCCGGTTCCGATCCTGATCCCGTGCCACCG<br>TGTTGTTAACATCAACGGTACCGTTGGTGGTTACGAAGGTGGTCTGGCGGTTAAAG<br>AATGGCTGCTGGCGCACGAAGGTCACCGTCTGGGTAAACCGGGTCTGGGTGGTAC<br>CGGCGGTCTGGTTCCACGTGGCTCCGGAGGCTCTGCGGGTTCTGCAGCTGGTTCT<br>GAATTCATGCCAACAGCAGCTTCTGCAACGGAATCAGCAATTGAAGACACACCAGC<br>TCCTGCCCGCCCTGAGGTTGATGGCCGCACAAAACCAAAGCGCCAACCACGCTAT<br>CATGTTGTACTCTGGGACGACGACGATCATACATATCAATACGTTGTGGTTATGCTCC<br>GAAGTTTGTTCGGCCATCCACCCGAACGCGGTTACCGCCTCGCAAAAGAGGTTGA<br>CACTCAAGGACGCGTGATTGTATTGACAACGACTCGCGAACATGCCGAGCTTAAGC<br>GCGACCAGATCCATGCATTCGGGCGAGATCGCCTTCTTGCACGCAGTAAAGGTTCG<br>ATGAAGGCGTCCATCGAGGCCGAAGAGGGCGGTGGCAGCGGTGGCGGTAGCGGC<br>GGTGGCAGCGGCGGCCTGAACGATATTTTTGAAGCGCAGAAAATTGAATGGCATGA<br>AGATTATAAAGATCATGATGGTGATTATAAAGATCACGATATCGACTACAAAGACGATG<br>ACGACAAGTGA
L68M,<br>V72M	ACCCGGCATGACAGGAGGATCGAGGCCTCTTAATACGACTCACTATAGGGGAATTG<br>TGAGCGGATAACAATTCCCCTCTAGAAATAATTTTGTTTAACTTTAAGAAGGAGATATA<br>CATATGGACAAAGACTGCGAAATGAAACGTACCACCCTGGACTCTCCGCTGGGTAA<br>ACTGGAACTGTCTGGTTGCGAACAGGGTCTGCACGAAATCATCTTCCTGGGTAAAG<br>GTACCTCTGCGGCGGACGCGGTTGAAGTTCCCGCACCGGCTGCGGTTCTGGGTG<br>GTCCGGAACCGCTGATGCAGGCGACCGCGTGGCTGAACGCGTACTTCCACCAGC<br>CGGAAGCGATCGAAGAATTCCCGGTTCCGGCGCTGCACCACCCGGTTTTCCAGCA<br>GGAATCTTTCACCCGTCAGGTCTTGTGGAAACTGCTGAAAGTTGTTAAATTCGGTGA<br>AGTTATCTCTTACTCTCACCTGGCGGCGCTGGCGGGTAACCCGGCAGCGACCGCG<br>GCAGTTAAAACCGCGCTGTCTGGTAACCCGGTTCCGATCCTGATCCCGTGCCACCG<br>TGTTGTTAACATCAACGGTACCGTTGGTGGTTACGAAGGTGGTCTGGCGGTTAAAG<br>AATGGCTGCTGGCGCACGAAGGTCACCGTCTGGGTAAACCGGGTCTGGGTGGTAC<br>CGGCGGTCTGGTTCCACGTGGCTCCGGAGGCTCTGCGGGTTCTGCAGCTGGTTCT<br>GAATTCATGCCAACAGCAGCTTCTGCAACGGAATCAGCAATTGAAGACACACCAGC<br>TCCTGCCCGCCCTGAGGTTGATGGCCGCACAAAACCAAAGCGCCAACCACGCTAT<br>CATGTTGTACTCTGGGACGACGACGATCATACATATCAATACGTTGTGGTTATGCTCC<br>AAAGTTTGTTCGGCCATCCACCCGAACGCGGTTACCGCATGGCAAAAGAGATGGAC<br>ACTCAAGGACGCGTGATTGTATTGACAACGACTCGCGAACATGCCGAGCTTAAGCG<br>CGACCAGATCCATGCATTCGGGTATGATCGCCTTCTTGCACGCAGTAAAGGTTCGAT<br>GAAGGCGTCCATCGAGGCCGAAGAGGGCGGTGGCAGCGGTGGCGGTAGCGGCG<br>GTGGCAGCGGCGGCCTGAACGATATTTTTGAAGCGCAGAAAATTGAATGGCATGAA

	GATTATAAAGATCATGATGGTGATTATAAAGATCACGATATCGACTACAAAGACGATGA<br>CGACAAGTGA
L68M, Y100R	ACCCGGCATGACAGGAGGATCGAGGCCTCTTAATACGACTCACTATAGGGGAATTG<br>TGAGCGGATAACAATTCCCCTCTAGAAATAATTTTGTTTAACTTTAAGAAGGAGATATA<br>CATATGGACAAAGACTGCGAAATGAAACGTACCACCCTGGACTCTCCGCTGGGTAA<br>ACTGGAACTGTCTGGTTGCGAACAGGGTCTGCACGAAATCATCTTCCTGGGTAAAG<br>GTACCTCTGCGGCGGACGCGGTTGAAGTTCCCGCACCGGCTGCGGTTCTGGGTG<br>GTCCGGAACCGCTGATGCAGGCGACCGCGTGGCTGAACGCGTACTTCCACCAGC<br>CGGAAGCGATCGAAGAATTCCCGGTTCCGGCGCTGCACCACCCGGTTTTCCAGCA<br>GGAATCTTTCACCCGTCAGGTCTTGTGGAAACTGCTGAAAGTTGTTAAATTCGGTGA<br>AGTTATCTCTTACTCTCACCTGGCGGCGCTGGCGGGTAACCCGGCAGCGACCGCG<br>GCAGTTAAAACCGCGCTGTCTGGTAACCCGGTTCCGATCCTGATCCCGTGCCACCG<br>TGTTGTTAACATCAACGGTACCGTTGGTGGTTACGAAGGTGGTCTGGCGGTTAAAG<br>AATGGCTGCTGGCGCACGAAGGTCACCGTCTGGGTAAACCGGGTCTGGGTGGTAC<br>CGGCGGTCTGGTTCCACGTGGCTCCGGAGGCTCTGCGGGTTCTGCAGCTGGTTCT<br>GAATTCATGCCAACAGCAGCTTCTGCAACGGAATCAGCAATTGAAGACACACCAGC<br>TCCTGCCCGCCCTGAGGTTGATGGCCGCACAAAACCAAAGCGCCAACCACGCTAT<br>CATGTTGTACTCTGGGACGACGACGATCATACATATCAATACGTTGTGGTTATGCTCC<br>AAAGTTTGTTCGGCCATCCACCCGAACGCGGTTACCGCATGGCAAAAGAGGTTGAC<br>ACTCAAGGACGCGTGATTGTATTGACAACGACTCGCGAACATGCCGAGCTTAAGCG<br>CGACCAGATCCATGCATTCGGGCGAGATCGCCTTCTTGCACGCAGTAAAGGTTCGA<br>TGAAGGCGTCCATCGAGGCCGAAGAGGGCGGTGGCAGCGGTGGCGGTAGCGGC<br>GGTGGCAGCGGCGGCCTGAACGATATTTTTGAAGCGCAGAAAATTGAATGGCATGA<br>AGATTATAAAGATCATGATGGTGATTATAAAGATCACGATATCGACTACAAAGACGATG<br>ACGACAAGTGA
V72M, Y100R	ACCCGGCATGACAGGAGGATCGAGGCCTCTTAATACGACTCACTATAGGGGAATTG<br>TGAGCGGATAACAATTCCCCTCTAGAAATAATTTTGTTTAACTTTAAGAAGGAGATATA<br>CATATGGACAAAGACTGCGAAATGAAACGTACCACCCTGGACTCTCCGCTGGGTAA<br>ACTGGAACTGTCTGGTTGCGAACAGGGTCTGCACGAAATCATCTTCCTGGGTAAAG<br>GTACCTCTGCGGCGGACGCGGTTGAAGTTCCCGCACCGGCTGCGGTTCTGGGTG<br>GTCCGGAACCGCTGATGCAGGCGACCGCGTGGCTGAACGCGTACTTCCACCAGC<br>CGGAAGCGATCGAAGAATTCCCGGTTCCGGCGCTGCACCACCCGGTTTTCCAGCA<br>GGAATCTTTCACCCGTCAGGTCTTGTGGAAACTGCTGAAAGTTGTTAAATTCGGTGA<br>AGTTATCTCTTACTCTCACCTGGCGGCGCTGGCGGGTAACCCGGCAGCGACCGCG<br>GCAGTTAAAACCGCGCTGTCTGGTAACCCGGTTCCGATCCTGATCCCGTGCCACCG<br>TGTTGTTAACATCAACGGTACCGTTGGTGGTTACGAAGGTGGTCTGGCGGTTAAAG<br>AATGGCTGCTGGCGCACGAAGGTCACCGTCTGGGTAAACCGGGTCTGGGTGGTAC<br>CGGCGGTCTGGTTCCACGTGGCTCCGGAGGCTCTGCGGGTTCTGCAGCTGGTTCT<br>GAATTCATGCCAACAGCAGCTTCTGCAACGGAATCAGCAATTGAAGACACACCAGC<br>TCCTGCCCGCCCTGAGGTTGATGGCCGCACAAAACCAAAGCGCCAACCACGCTAT<br>CATGTTGTACTCTGGGACGACGACGATCATACATATCAATACGTTGTGGTTATGCTCC<br>AAAGTTTGTTCGGCCATCCACCCGAACGCGGTTACCGCCTCGCAAAAGAGATGGAC<br>ACTCAAGGACGCGTGATTGTATTGACAACGACTCGCGAACATGCCGAGCTTAAGCG<br>CGACCAGATCCATGCATTCGGGCGAGATCGCCTTCTTGCACGCAGTAAAGGTTCGA<br>TGAAGGCGTCCATCGAGGCCGAAGAGGGCGGTGGCAGCGGTGGCGGTAGCGGC<br>GGTGGCAGCGGCGGCCTGAACGATATTTTTGAAGCGCAGAAAATTGAATGGCATGA<br>AGATTATAAAGATCATGATGGTGATTATAAAGATCACGATATCGACTACAAAGACGATG<br>ACGACAAGTGA
Q55R, L68M, V72M	ACCCGGCATGACAGGAGGATCGAGGCCTCTTAATACGACTCACTATAGGGGAATTG<br>TGAGCGGATAACAATTCCCCTCTAGAAATAATTTTGTTTAACTTTAAGAAGGAGATATA<br>CATATGGACAAAGACTGCGAAATGAAACGTACCACCCTGGACTCTCCGCTGGGTAA<br>ACTGGAACTGTCTGGTTGCGAACAGGGTCTGCACGAAATCATCTTCCTGGGTAAAG<br>GTACCTCTGCGGCGGACGCGGTTGAAGTTCCCGCACCGGCTGCGGTTCTGGGTG<br>GTCCGGAACCGCTGATGCAGGCGACCGCGTGGCTGAACGCGTACTTCCACCAGC<br>CGGAAGCGATCGAAGAATTCCCGGTTCCGGCGCTGCACCACCCGGTTTTCCAGCA<br>GGAATCTTTCACCCGTCAGGTCTTGTGGAAACTGCTGAAAGTTGTTAAATTCGGTGA<br>AGTTATCTCTTACTCTCACCTGGCGGCGCTGGCGGGTAACCCGGCAGCGACCGCG<br>GCAGTTAAAACCGCGCTGTCTGGTAACCCGGTTCCGATCCTGATCCCGTGCCACCG

	TGTTGTTAACATCAACGGTACCGTTGGTGGTTACGAAGGTGGTCTGGCGGTTAAAG<br>AATGGCTGCTGGCGCACGAAGGTCACCGTCTGGGTAAACCGGGTCTGGGTGGTAC<br>CGGCGGTCTGGTTCCACGTGGCTCCGGAGGCTCTGCGGGTTCTGCAGCTGGTTCT<br>GAATTCATGCCAACAGCAGCTTCTGCAACGGAATCAGCAATTGAAGACACACCAGC<br>TCCTGCCCGCCCTGAGGTTGATGGCCGCACAAAACCAAAGCGCCAACCACGCTAT<br>CATGTTGTACTCTGGGACGACGACGATCATACATATCAATACGTTGTGGTTATGCTCC<br>GAAGTTTGTTCGGCCATCCACCCGAACGCGGTTACCGCATGGCAAAAGAGATGGA<br>CACTCAAGGACGCGTGATTGTATTGACAACGACTCGCGAACATGCCGAGCTTAAGC<br>GCGACCAGATCCATGCATTCGGGTATGATCGCCTTCTTGCACGCAGTAAAGGTTCG<br>ATGAAGGCGTCCATCGAGGCCGAAGAGGGCGGTGGCAGCGGTGGCGGTAGCGGC<br>GGTGGCAGCGGCGGCCTGAACGATATTTTTGAAGCGCAGAAAATTGAATGGCATGA<br>AGATTATAAAGATCATGATGGTGATTATAAAGATCACGATATCGACTACAAAGACGATG<br>ACGACAAGTGA
Q55R, L68M, Y100R	ACCCGGCATGACAGGAGGATCGAGGCCTCTTAATACGACTCACTATAGGGGAATTG<br>TGAGCGGATAACAATTCCCCTCTAGAAATAATTTTGTTTAACTTTAAGAAGGAGATATA<br>CATATGGACAAAGACTGCGAAATGAAACGTACCACCCTGGACTCTCCGCTGGGTAA<br>ACTGGAACTGTCTGGTTGCGAACAGGGTCTGCACGAAATCATCTTCCTGGGTAAAG<br>GTACCTCTGCGGCGGACGCGGTTGAAGTTCCCGCACCGGCTGCGGTTCTGGGTG<br>GTCCGGAACCGCTGATGCAGGCGACCGCGTGGCTGAACGCGTACTTCCACCAGC<br>CGGAAGCGATCGAAGAATTCCCGGTTCCGGCGCTGCACCACCCGGTTTTCCAGCA<br>GGAATCTTTCACCCGTCAGGTCTTGTGGAAACTGCTGAAAGTTGTTAAATTCGGTGA<br>AGTTATCTCTTACTCTCACCTGGCGGCGCTGGCGGGTAACCCGGCAGCGACCGCG<br>GCAGTTAAAACCGCGCTGTCTGGTAACCCGGTTCCGATCCTGATCCCGTGCCACCG<br>TGTTGTTAACATCAACGGTACCGTTGGTGGTTACGAAGGTGGTCTGGCGGTTAAAG<br>AATGGCTGCTGGCGCACGAAGGTCACCGTCTGGGTAAACCGGGTCTGGGTGGTAC<br>CGGCGGTCTGGTTCCACGTGGCTCCGGAGGCTCTGCGGGTTCTGCAGCTGGTTCT<br>GAATTCATGCCAACAGCAGCTTCTGCAACGGAATCAGCAATTGAAGACACACCAGC<br>TCCTGCCCGCCCTGAGGTTGATGGCCGCACAAAACCAAAGCGCCAACCACGCTAT<br>CATGTTGTACTCTGGGACGACGACGATCATACATATCAATACGTTGTGGTTATGCTCC<br>GAAGTTTGTTCGGCCATCCACCCGAACGCGGTTACCGCATGGCAAAAGAGGTTGA<br>CACTCAAGGACGCGTGATTGTATTGACAACGACTCGCGAACATGCCGAGCTTAAGC<br>GCGACCAGATCCATGCATTCGGGCGAGATCGCCTTCTTGCACGCAGTAAAGGTTCG<br>ATGAAGGCGTCCATCGAGGCCGAAGAGGGCGGTGGCAGCGGTGGCGGTAGCGGC<br>GGTGGCAGCGGCGGCCTGAACGATATTTTTGAAGCGCAGAAAATTGAATGGCATGA<br>AGATTATAAAGATCATGATGGTGATTATAAAGATCACGATATCGACTACAAAGACGATG<br>ACGACAAGTGA
Q55R, V72M, Y100R	ACCCGGCATGACAGGAGGATCGAGGCCTCTTAATACGACTCACTATAGGGGAATTG<br>TGAGCGGATAACAATTCCCCTCTAGAAATAATTTTGTTTAACTTTAAGAAGGAGATATA<br>CATATGGACAAAGACTGCGAAATGAAACGTACCACCCTGGACTCTCCGCTGGGTAA<br>ACTGGAACTGTCTGGTTGCGAACAGGGTCTGCACGAAATCATCTTCCTGGGTAAAG<br>GTACCTCTGCGGCGGACGCGGTTGAAGTTCCCGCACCGGCTGCGGTTCTGGGTG<br>GTCCGGAACCGCTGATGCAGGCGACCGCGTGGCTGAACGCGTACTTCCACCAGC<br>CGGAAGCGATCGAAGAATTCCCGGTTCCGGCGCTGCACCACCCGGTTTTCCAGCA<br>GGAATCTTTCACCCGTCAGGTCTTGTGGAAACTGCTGAAAGTTGTTAAATTCGGTGA<br>AGTTATCTCTTACTCTCACCTGGCGGCGCTGGCGGGTAACCCGGCAGCGACCGCG<br>GCAGTTAAAACCGCGCTGTCTGGTAACCCGGTTCCGATCCTGATCCCGTGCCACCG<br>TGTTGTTAACATCAACGGTACCGTTGGTGGTTACGAAGGTGGTCTGGCGGTTAAAG<br>AATGGCTGCTGGCGCACGAAGGTCACCGTCTGGGTAAACCGGGTCTGGGTGGTAC<br>CGGCGGTCTGGTTCCACGTGGCTCCGGAGGCTCTGCGGGTTCTGCAGCTGGTTCT<br>GAATTCATGCCAACAGCAGCTTCTGCAACGGAATCAGCAATTGAAGACACACCAGC<br>TCCTGCCCGCCCTGAGGTTGATGGCCGCACAAAACCAAAGCGCCAACCACGCTAT<br>CATGTTGTACTCTGGGACGACGACGATCATACATATCAATACGTTGTGGTTATGCTCC<br>GAAGTTTGTTCGGCCATCCACCCGAACGCGGTTACCGCCTCGCAAAAGAGATGGA<br>CACTCAAGGACGCGTGATTGTATTGACAACGACTCGCGAACATGCCGAGCTTAAGC<br>GCGACCAGATCCATGCATTCGGGCGAGATCGCCTTCTTGCACGCAGTAAAGGTTCG<br>ATGAAGGCGTCCATCGAGGCCGAAGAGGGCGGTGGCAGCGGTGGCGGTAGCGGC<br>GGTGGCAGCGGCGGCCTGAACGATATTTTTGAAGCGCAGAAAATTGAATGGCATGA

	AGATTATAAAGATCATGATGGTGATTATAAAGATCACGATATCGACTACAAAGACGATG ACGACAAGTGA
L68M, V72M, Y100R	ACCCGGCATGACAGGAGGATCGAGGCCTCTTAATACGACTCACTATAGGGGAATTG TGAGCGGATAACAATTCCCCTCTAGAAATAATTTTGTTTAACTTTAAGAAGGAGATATA CATATGGACAAAGACTGCGAAATGAAACGTACCACCCTGGACTCTCCGCTGGGTAA ACTGGAACTGTCTGGTTGCGAACAGGGTCTGCACGAAATCATCTTCCTGGGTAAAG GTACCTCTGCGGCGGACGCGGTTGAAGTTCCCGCACCGGCTGCGGTTCTGGGTG GTCCGGAACCGCTGATGCAGGCGACCGCGTGGCTGAACGCGTACTTCCACCAGC CGGAAGCGATCGAAGAATTCCCGGTTCCGGCGCTGCACCACCCGGTTTTCCAGCA GGAATCTTTCACCCGTCAGGTCTTGTGGAAACTGCTGAAAGTTGTTAAATTCGGTGA AGTTATCTCTTACTCTCACCTGGCGGCGCTGGCGGGTAACCCGGCAGCGACCGCG GCAGTTAAAACCGCGCTGTCTGGTAACCCGGTTCCGATCCTGATCCCGTGCCACCG TGTTGTTAACATCAACGGTACCGTTGGTGGTTACGAAGGTGGTCTGGCGGTTAAAG AATGGCTGCTGGCGCACGAAGGTCACCGTCTGGGTAAACCGGGTCTGGGTGGTAC CGGCGGTCTGGTTCCACGTGGCTCCGGAGGCTCTGCGGGTTCTGCAGCTGGTTCT GAATTCATGCCAACAGCAGCTTCTGCAACGGAATCAGCAATTGAAGACACACCAGC TCCTGCCCGCCCTGAGGTTGATGGCCGCACAAAACCAAAGCGCCAACCACGCTAT CATGTTGTACTCTGGGACGACGACGATCATACATATCAATACGTTGTGGTTATGCTCC AAAGTTTGTTCGGCCATCCACCCGAACGCGGTTACCGCATGGCAAAAGAGATGGAC ACTCAAGGACGCGTGATTGTATTGACAACGACTCGCGAACATGCCGAGCTTAAGCG CGACCAGATCCATGCATTCGGGCGAGATCGCCTTCTTGCACGCAGTAAAGGTTCGA TGAAGGCGTCCATCGAGGCCGAAGAGGGCGGTGGCAGCGGTGGCGGTAGCGGC GGTGGCAGCGGCGGCCTGAACGATATTTTTGAAGCGCAGAAAATTGAATGGCATGA AGATTATAAAGATCATGATGGTGATTATAAAGATCACGATATCGACTACAAAGACGATG ACGACAAGTGA
Q55R, L68M, V72M, Y100R (ClpS-Opt)	ACCCGGCATGACAGGAGGATCGAGGCCTCTTAATACGACTCACTATAGGGGAATTG TGAGCGGATAACAATTCCCCTCTAGAAATAATTTTGTTTAACTTTAAGAAGGAGATATA CATATGGACAAAGACTGCGAAATGAAACGTACCACCCTGGACTCTCCGCTGGGTAA ACTGGAACTGTCTGGTTGCGAACAGGGTCTGCACGAAATCATCTTCCTGGGTAAAG GTACCTCTGCGGCGGACGCGGTTGAAGTTCCCGCACCGGCTGCGGTTCTGGGTG GTCCGGAACCGCTGATGCAGGCGACCGCGTGGCTGAACGCGTACTTCCACCAGC CGGAAGCGATCGAAGAATTCCCGGTTCCGGCGCTGCACCACCCGGTTTTCCAGCA GGAATCTTTCACCCGTCAGGTCTTGTGGAAACTGCTGAAAGTTGTTAAATTCGGTGA AGTTATCTCTTACTCTCACCTGGCGGCGCTGGCGGGTAACCCGGCAGCGACCGCG GCAGTTAAAACCGCGCTGTCTGGTAACCCGGTTCCGATCCTGATCCCGTGCCACCG TGTTGTTAACATCAACGGTACCGTTGGTGGTTACGAAGGTGGTCTGGCGGTTAAAG AATGGCTGCTGGCGCACGAAGGTCACCGTCTGGGTAAACCGGGTCTGGGTGGTAC CGGCGGTCTGGTTCCACGTGGCTCCGGAGGCTCTGCGGGTTCTGCAGCTGGTTCT GAATTCATGCCAACAGCAGCTTCTGCAACGGAATCAGCAATTGAAGACACACCAGC TCCTGCCCGCCCTGAGGTTGATGGCCGCACAAAACCAAAGCGCCAACCACGCTAT CATGTTGTACTCTGGGACGACGACGATCATACATATCAATACGTTGTGGTTATGCTCC GAAGTTTGTTCGGCCATCCACCCGAACGCGGTTACCGCATGGCAAAAGAGATGGA CACTCAAGGACGCGTGATTGTATTGACAACGACTCGCGAACATGCCGAGCTTAAGC GCGACCAGATCCATGCATTCGGGCGAGATCGCCTTCTTGCACGCAGTAAAGGTTCG ATGAAGGCGTCCATCGAGGCCGAAGAGGGCGGTGGCAGCGGTGGCGGTAGCGGC GGTGGCAGCGGCGGCCTGAACGATATTTTTGAAGCGCAGAAAATTGAATGGCATGA AGATTATAAAGATCATGATGGTGATTATAAAGATCACGATATCGACTACAAAGACGATG ACGACAAGTGA
