# Supplementary material for: Multiplexed single-molecule analysis of barcoded protein variants
Source: Nat Commun. 2026 Oct 12;17:11560. doi: 10.1038/s41467-026-78581-y (PMC13748599; doi:10.1038/s41467-026-78581-y)
Supplement: Supplementary file 2 [file 41467_2026_78581_MOESM2_ESM.pdf]

nature portfolio

Peer Review File

Multiplexed single-molecule analysis of barcoded protein variants

Corresponding Author: Dr Lisanne Spenkelink

This file contains all reviewer reports in order by version, followed by all author rebuttals in order by version.

Version 0:

Reviewer comments:

Reviewer #2

(Remarks to the Author)
This study presents a technically well-executed and conceptually interesting approach in which protein variants are barcoded with DNA sequences and analysed using single-molecule fluorescence. The work addresses an important challenge in the field, namely, how to access functional heterogeneity across multiple protein variants in parallel at the single-molecule level. In the view of this referee, the manuscript should primarily be evaluated as a methodological contribution; in that context, the central question is the extent to which the platform provides a robust and scalable advance over existing multiplexed single-molecule approaches. Overall, this referee recommends the publication of this work but with major editorial corrections.

Major concerns:
1. The main concern relates to the practical scalability of the method. A more realistic and quantitative assessment of achievable throughput is necessary, or alternatively the claims regarding scalability should be moderated and the applicable regime of the method should be clearly defined as follows:
1a. While the encoding scheme based on probe identity, fluorophore combinations, and kinetic signatures is in principle extensible, the current implementation appears to be strongly limited by acquisition time. In the current study the imaging time for barcode detection already seems to be 60 minutes per FOV (2 rounds, 3 probes, 10 min/round/probe). Barcode identification requires multiple rounds of imaging with several probes, leading to a total acquisition time that substantially exceeds that of the functional measurement itself. As the number of variants increases, additional rounds and fields of view will be required, and it is not clear how this scales in practice.
1b. In addition, each protein variant must be individually synthesised and barcoded, which introduces further constraints on library size and preparation time. At present, the manuscript provides only a combinatorial estimate of barcode capacity, without addressing these experimental bottlenecks.

2. The general concept of linking molecular identity to single-molecule measurements using DNA barcodes, including DNA-PAINT-based readout, has been explored in several previous studies. The present work extends this concept to protein–peptide interactions, which is a meaningful application, but the manuscript does not sufficiently acknowledge or contrast prior approaches. A more balanced discussion would help clarify what is specifically enabled here. Following are a few examples.
https://pubs.acs.org/doi/10.1021/acs.nanolett.0c04502
https://doi.org/10.1016/j.bpj.2018.08.013
https://pubs.acs.org/doi/10.1021/jacs.1c06195
https://doi.org/10.1101/2022.02.27.482175

3. The Discussion would benefit from being more focused. At present, it contains several speculative extensions and future applications that are not directly supported by the data. Reducing this section and instead emphasising the demonstrated capabilities and current limitations of the platform would improve the overall balance of the manuscript.

4. Figure 1. While panel 1 highlights the aim of the manuscript, it is immediately followed by the workflow of the barcoding scheme only. Consider placing Figure 3a in Figure 1 to give an overview of the entire workflow (protein immmobilisation peptide binding barcode decoding) early in the manuscript.

5. It is not clear how 16 variants were selected and thus how this covers “all possible evolutionary trajectories”.

6. Some aspects of the data interpretation and presentation would also benefit from clarification. Check the minor comments.

Minor points
22 References 1-6: Four out of six are their own papers.
33 Not “attempted”, but “succeeded”
33 A wrong reference
101 It would take twice long to image.
111 Does the error rate depend on the average dwell time? If so, for some combinations the error rate may be higher than others. It is advised to report the error rates for the different kinetics separately.
111 How is this 5% calculated? Is it a fraction of the number of molecules?
112 In Figure 2 there are two probe sites per molecule, but in extended data figure 2 it goes back to a single probe site per molecule, with 18 different possibilities. And then in Figure 3 there are two probe sites again. This is confusing. Note that the error rates for having a single probe will be different compared to having a double probe per molecule.
160 Do the two different probe oligos hybridize to two different positions or a single position on the DNA?
165 This was already known from earlier (not single-molecule) studies.
172 Make it clear what new things we expect and learn here.
181 This conflicts with the statement the bimodal distribution of the WT came from different conformations, and that the mutated versions of the protein removed one of the conformations. Please clarify.
197 Are these the off state, or on state? The times, 7 and 1.5 seconds, seem to correspond to the upper panel in figure 3c, which is the on-dwell time.
209 Concerning the time to measure the barcodes, it seems like barcode identification takes 10 minutes per colour/probe per round, so in total 10x3x2 = 60 minutes per FOV, while the actual measurement of the peptide interaction (phenotype reaction) takes 10 minutes per FOV. So, measuring the 16 protein variants separately would take 10*16 = 160 minutes, while measuring in parallel would take 60+10=70 minutes per FOV (assuming 1 FOV, which is probably not enough). So it seems the parallel setup offers a 160/70 = 2.3 times reduction in measurement time. And if we need two FOVs, this reduces to 160/(2*70) = 1.1. If this is correct, please mention this in the text. If this is incorrect, then please clarify in the text.
211 It is not clear why the reduction in acquisition time would be higher for replicates.
219 Clarify what indices means here
248 Not entirely clear. Protein functional analysis of individual protein sequences then?
389 Please justify the use of Alphafold while there is already a PDB structure.
Fig 1b: Suggestion: transient hybridization (instead of annealing)
Fig 2a: In the figure it looks as if the main strand is not continuous (after nicking). But it is expected that only one of the strands is cut and the other one remains completely intact.
Fig 2b: Scalebar not defined
Fig 3: An example trace of the peptide binding to the ClpS would be helpful.
Fig 3c: Is it only 2 modes or 3 modes? For some it looks like three populations. If a molecule switches conformation within a trajectory, then taking the mean times would result in intermediate values.
Extended data Fig 1: How is the clustering done when the population is not present? Why are there dark purple molecules in between the blue and green populations.

Reviewer #3

(Remarks to the Author)
I have read the paper ‘Multiplexed single-molecule analysis of barcoded protein variants’ by Mueller et al. The paper described a DNA barcoding procedure that allows for immobilized proteins to be positively identified during a single-molecule microscopy experiment, thus allowing for multiple protein variants to be characterized within the same experiment. The authors then use variants of plantomycetes ClpS previously reported in the literature as a proof of principle experiment. Their barcoding system is novel, well-characterized, and thoroughly analyzed. I would recommend this paper for publication.

In paragraph 4 of the section titled ‘Main’, the authors write that the experiments will demonstrate “…differences in kinetic behavior driven primarily by the relative abundance of each kinetic phenotype…”. I think this statement suggests that the paper will be comparing the unique bimodal kinetics of each mutant, rather than comparing the unimodal kinetics caused by a single mutant against the bimodal kinetics of the each other variant. What the paper accomplishes is itself good, but I think the introduction would be clearer without this sentence accidentally giving readers incorrect expectations.

I would suggest that at the sentence “Combining the colour of the probe with the dwell time of its binding, allows the identification of hundreds of thousands of protein variants in the same assay” should be rewritten “…binding allows for…” and include a citation to ‘A theoretical analysis of single molecule protein sequencing via weak binding spectra’ by Rodriques, Marblestone, & Boyden.

In paragraph 1 of the section ‘Main’, the following edit be made: “…revealing heterogeneous behaviors that are obscured in bulk measurements and transform[ing] our ability to probe the dynamics of biomolecular interactions.”

This work employs a ClpS-SNAP fusion construct. Because of the small size of ClpS and the conformational changes to the N and C terminals reported in the literature upon binding, the SNAP domain could arguably affect binding. A comparison of ClpS binding with and without the snap tag by an orthogonal method such as BLI or SPR would strengthen this paper.

In paragraph 2 of the section Main, the claim is made "In typical single-molecule imaging, the number of distinguishable species is constrained by the limited number of fluorophores that can be used simultaneously." I agree with this claim, but feel the paper would be strengthened if a citation more fully demonstrating this limitation could be provided.

I would suggest that in addition to citations 18 and 19 of Tullman et al., a citation should be included for 'Contributions from ClpS surface residues in modulating N-terminal peptide binding and their implications for NAAB development' by Callahan et al.

The authors say in the first paragraph of the discussion section that they are working with variants of planctomycetes ClpS. This information should be clarified before the experimental results and a standard terminology ("ClpS") be established for the rest of the paper.

Paragraph 3 of the section Discussion brings up the possible role of the ClpS dynamic regime being altered during ClpA binding. The authors are right to cite reference 26, but should also consider citing and incorporating points from 'AAA+ protease-adaptor structures reveal altered conformations and ring specialization' by Kim et al. and 'A single amino acid substitution alters ClpS2 binding specificity' by Bergonzo et al.

Reviewer #4

(Remarks to the Author)
I co-reviewed this manuscript with one of the reviewers who provided the listed reports. This is part of the Nature Communications initiative to facilitate training in peer review and to provide appropriate recognition for Early Career Researchers who co-review manuscripts.

Version 1:

Reviewer comments:

Reviewer #2

(Remarks to the Author)
The authors have clarified several points raised in the previous review. It is now clearer that multiple indices can be imaged without necessarily requiring sequential measurements for each variant. However, I have two remaining concerns that should be addressed before publication.

First, the calculation of the theoretical barcode capacity appears to be inconsistent. The authors state that they calculate the number of possible barcodes using the binomial coefficient with replacement, but the explicit formula is not shown. Using the standard formula for combinations with replacement, C(n + r - 1, r), and excluding the all-no-signal case, the expected number of usable barcodes would be C(n + r - 1, r) - 1, where n is the number of distinguishable signal states per index, including the no-binding state, and r is the number of indices. If three probes with three kinetic signatures are used, there are 9 observable signal classes plus one no-binding class, giving n = 10. This gives 9, 54, 219, and 5,004 possible barcodes for 1, 2, 3, and 6 indices, respectively. Similarly, for six probes with three kinetic signatures, there are 18 observable signal classes plus one no-binding class, giving n = 19. The corresponding numbers are therefore 18, 189, 1,329, and 134,595. These values appear to differ from the numbers currently stated in the manuscript, including 270, >20,000, and >300,000 barcodes. The authors should therefore check and correct these numbers, or explain more explicitly if a different counting scheme is intended.

Second, the practical measurement time for large libraries remains insufficiently quantified. The authors now state that 16 variants can be measured in parallel in 35 min, compared with 80 min if measured individually, corresponding to a 2.3-fold reduction in imaging time. This is useful. However, the extrapolation to much larger libraries remains unclear. For example, if approximately 2,000 molecules can be resolved per field of view, then a library of 134,595 variants would require roughly 67 fields of view merely to observe one molecule per variant on average. This would correspond to approximately 67 hours of acquisition time at 1 hour per field of view, and substantially longer if multiple molecules per variant are needed for reliable statistics. Even 10 molecules per variant would require approximately 28 days of measurement time under these assumptions. The manuscript should therefore provide a more realistic quantitative estimate of the experimentally accessible library size, or moderate the claims about scalability accordingly.

Reviewer #3

(Remarks to the Author)
I am satisfied with the revisions and recommend publication.

Reviewer #4

(Remarks to the Author)
I co-reviewed this manuscript with one of the reviewers who provided the listed reports. This is part of the Nature Communications initiative to facilitate training in peer review and to provide appropriate recognition for Early Career

Researchers who co-review manuscripts.

Version 2:

Reviewer comments:

Reviewer #2

(Remarks to the Author)
The authors have clarified the basis of their barcode-capacity calculation. The theoretical capacity is likely overestimated because some barcode–kinetic combinations are not distinguishable, especially when the number of indices increases. Nevertheless, as the manuscript clearly states how the calculation was performed and avoids overstating the practical scalability, I am satisfied with the revision and recommend publication.

Open Access This Peer Review File is licensed under a Creative Commons Attribution 4.0 International License, which permits use, sharing, adaptation, distribution and reproduction in any medium or format, as long as you give appropriate credit to the original author(s) and the source, provide a link to the Creative Commons license, and indicate if changes were made.
In cases where reviewers are anonymous, credit should be given to 'Anonymous Referee' and the source.
The images or other third party material in this Peer Review File are included in the article's Creative Commons license, unless indicated otherwise in a credit line to the material. If material is not included in the article's Creative Commons license and your intended use is not permitted by statutory regulation or exceeds the permitted use, you will need to obtain permission directly from the copyright holder.
To view a copy of this license, visit https://creativecommons.org/licenses/by/4.0/

We thank the reviewers for their positive assessment of our work and thoughtful feedback. Please find a point-by-point response to the reviewer comments below.

Reviewer #2 (Remarks to the Author):

This study presents a technically well-executed and conceptually interesting approach in which protein variants are barcoded with DNA sequences and analysed using single-molecule fluorescence. The work addresses an important challenge in the field, namely, how to access functional heterogeneity across multiple protein variants in parallel at the single-molecule level. In the view of this referee, the manuscript should primarily be evaluated as a methodological contribution; in that context, the central question is the extent to which the platform provides a robust and scalable advance over existing multiplexed single-molecule approaches. Overall, this referee recommends the publication of this work but with major editorial corrections.

Major concerns:

1. The main concern relates to the practical scalability of the method. A more realistic and quantitative assessment of achievable throughput is necessary, or alternatively the claims regarding scalability should be moderated and the applicable regime of the method should be clearly defined as follows:

1a. While the encoding scheme based on probe identity, fluorophore combinations, and kinetic signatures is in principle extensible, the current implementation appears to be strongly limited by acquisition time. In the current study the imaging time for barcode detection already seems to be 60 minutes per FOV (2 rounds, 3 probes, 10 min/round/probe). Barcode identification requires multiple rounds of imaging with several probes, leading to a total acquisition time that substantially exceeds that of the functional measurement itself. As the number of variants increases, additional rounds and fields of view will be required, and it is not clear how this scales in practice.

Increasing the number of variants does not per se require sequential measurements. Within a single round, increasing the number of indices expands the number of identifiable barcodes from 54 (2 indices) to 270 (3 indices) and 20,412 (6 indices), without increasing acquisition time per round. Adding a second round (total imaging time ~60 minutes) allows use of 6 probes, increasing capacity to 189 (2 indices), 1,512 (3 indices), and 336,798 (6 indices). In practice, the number of variants that can be analysed in a single experiment is limited by the field of view, which in our setup typically contains ~2,000 molecules.

1b. In addition, each protein variant must be individually synthesised and barcoded, which introduces further constraints on library size and preparation time. At present, the manuscript provides only a combinatorial estimate of barcode capacity, without addressing these experimental bottlenecks.

In the current study, protein variants were generated and barcoded individually. Expression volumes of 15µL produced ample amounts of barcoded protein without the necessity of purification. Commercially available as well as open-source liquid handling platforms routinely enable the parallel processing of hundreds to thousands of samples in 96-, 384 or even 1536-well plates. This allows for straightforward scaling to thousands of variants (ref1ref2)In addition to this, our barcoding strategy is based on SNAP-tag chemistry coupled to in vitro expression, which is compatible with droplet-based workflows, enabling large libraries to be expressed and barcoded in parallel (refs 21, 30, 39–41).

To clarify both points, we have changed the paragraph in the discussion. The text now reads: "A key advantage of simultaneous measurement is the elimination of experiment-to-experiment variation, which is critical in single-molecule assays, where experimental conditions can be difficult to standardise37, 38. In addition, parallelisation reduces measurement time. In our implementation, measuring 16 variants in parallel requires 35 minutes of imaging time (30 min barcode acquisition + 5 min phenotype), compared to 80 minutes if measured individually, corresponding to an approximately 2.3-fold reduction. Beyond measurement efficiency, our workflow also offers practical advantages. Gene variants were ordered as synthetic eBlocks, eliminating the need for cloning and simplifying construct preparation. Proteins were expressed via cell-free expression, and barcode coupling occurred in the same reaction through SNAP-tag chemistry, avoiding extra purification steps. These features streamline assay setup and make the method well-suited to rapid, iterative experiments."

2. The general concept of linking molecular identity to single-molecule measurements using DNA barcodes, including DNA-PAINT-based readout, has been explored in several previous studies. The present work extends this concept to protein–peptide interactions, which is a meaningful application, but the manuscript does not sufficiently acknowledge or contrast prior approaches. A more balanced discussion would help clarify what is specifically enabled here. Following are a few examples.

https://pubs.acs.org/doi/10.1021/acs.nanolett.0c04502

https://doi.org/10.1016/j.bpj.2018.08.013

https://pubs.acs.org/doi/10.1021/jacs.1c06195

https://doi.org/10.1101/2022.02.27.482175

We agree and thank the reviewer for the suggestion. We have added the following text and references (excluding the Andrews et al. bioRxiv manuscript) to the Discussion: "Previous studies have established the use of DNA barcodes, including DNA-PAINT-based approaches, to enable multiplexed identification of molecular species at the single-molecule level. These approaches have primarily focused on spatial localisation, counting, or structural characterisation, rather than linking molecular identity to functional measurements[46-48]"

3. The Discussion would benefit from being more focused. At present, it contains several speculative extensions and future applications that are not directly supported by the data. Reducing this section and instead emphasising the demonstrated capabilities and current limitations of the platform would improve the overall balance of the manuscript.

We have revised the Discussion to reduce speculative statements and to place greater emphasis on the demonstrated capabilities of the platform. In particular, we removed forward-looking statements and streamlined sections on potential future applications

4. Figure 1. While panel 1 highlights the aim of the manuscript, it is immediately followed by the workflow of the barcoding scheme only. Consider placing Figure 3a in Figure 1 to give an overview of the entire workflow (protein immmobilisation □ peptide binding □ barcode decoding) early in the manuscript.

Figure 1a serves as a concept figure, illustrating the aims of the study rather than the experimental workflow. Introducing workflow details at this stage would be premature, as the individual components (protein immobilisation, SNAP-tag labelling, barcode design) have not yet been described. Figure 3a provides the complete workflow overview in the appropriate

context. Instead, to improve clarity, we have revised Figure 1 by adding visual connectors highlighting where Fig 1b sits within the workflow, and we have changed the header of Fig. 1b to ‘ single-molecule barcoding workflow’.

5. It is not clear how 16 variants were selected and thus how this covers “all possible evolutionary trajectories”.

We apologise for the lack of clarity. We refer to an evolutionary trajectory as every possible order in which the mutations of the studied variant can be acquired (see Fig. 3b.). This is significant, because we not only investigate the impact of every single-point mutation, but rather the impact of every point mutation in every possible context of all other point-mutations. This allows the identification of mutational crosstalk and allosteric effects. We have revised the text in the discussion to: “These variants cover all possible combinations of the four mutations between wild-type ClpS and ClpS-Opt, capturing all possible mutational intermediates between these states (Fig. 3b).”

6. Some aspects of the data interpretation and presentation would also benefit from clarification. Check the minor comments.

We have addressed all minor comments as detailed below.

Minor points

22 References 1-6: Four out of six are their own papers.

We have added the following additional references to provide a more balanced context: PMIDs 26097470, 29282758, 29197672, 32228033, 33995902, 9836635

33 Not “attempted”, but “succeeded”

We have changed the text to: “Some recent approaches, such as MUSCLE[19] and SPARXS[20], have increased throughput using indexing strategies….”

33 A wrong reference

The reference has been corrected.

101 It would take twice long to image.

We have clarified how acquisition time scales with the number of indices in the Discussion (see response to major comment 1).

111 Does the error rate depend on the average dwell time? If so, for some combinations the error rate may be higher than others. It is advised to report the error rates for the different kinetics separately.

We agree that, in principle, classification accuracy could depend on dwell time. However, the kinetic states used in this study were explicitly selected to balance this effect. Fast-binding states generate a large number of events within the observation window, providing strong statistical power for accurate identification. In contrast, slow-binding states produce fewer events, but each event carries high information content due to the exponential dependence of dwell time on binding kinetics, making even a small number of long-lived events highly discriminative. As a result, the effective classification accuracy is comparable across the different kinetic states, and no systematic bias in error rate with dwell time was observed and we have therefore reported a global classification accuracy. Misclassification is primarily driven by nonspecific background probe binding events and spatial overlap between barcodes

111 How is this 5% calculated? Is it a fraction of the number of molecules?

We have clarified how the accuracy is calculated. Specifically, the reported 95% accuracy corresponds to the fraction of correctly identified molecules relative to the total number of classified molecules. The text now reads: "We then counted how often the algorithm identified a barcode that was not actually present (Extended Data Fig. 1). Classification accuracy was calculated as the fraction of correctly identified molecules over the total number of classified molecules."

112 In Figure 2 there are two probe sites per molecule, but in extended data figure 2 it goes back to a single probe site per molecule, with 18 different possibilities. And then in Figure 3 there are two probe sites again. This is confusing. Note that the error rates for having a single probe will be different compared to having a double probe per molecule.

We apologise for the confusion. Extended Data Fig. 2 shows single indices for simplicity, whereas the same workflow applies to multi-index barcodes used in Figures 2 and 3. We have clarified this in the legend for Extended Data Fig. 2. Misclassification rates were calculated using 2-index barcodes.

160 Do the two different probe oligos hybridize to two different positions or a single position on the DNA?

The two probe oligos hybridise to two distinct positions on the DNA. We have clarified this in the text. The text now reads: " Following simultaneous immobilisation of all 16 variants, two spectrally distinct probe oligos were introduced to enable barcode identification (Fig. 3a), each hybridising to a distinct index."

165 This was already known from earlier (not single-molecule) studies.

We have added the relevant reference to reflect this (PMID: 36227977).

172 Make it clear what new things we expect and learn here.

We have clarified the expected outcomes and the insight gained from these data. Specifically, one might expect mutations to produce a uniform shift in dwell times across all events; however, we observe that the dwell time modes remain unchanged and that the differences arise from shifts in the relative abundance of two kinetic subpopulations.

181 This conflicts with the statement the bimodal distribution of the WT came from different conformations, and that the mutated versions of the protein removed one of the conformations. Please clarify.

We have revised the text to include our interpretation of the link between the molecular dynamics results and the experimentally observed kinetic behaviour. The text now reads: "The simulations revealed altered van der Waals (Fig. 3e) and electrostatic interactions (Extended Data Fig. 6) with the N-terminal leucine residue of the peptide. This may explain the shift in subpopulation abundance observed experimentally, with V72M altering peptide interactions."

197 Are these the off state, or on state? The times, 7 and 1.5 seconds, seem to correspond to the upper panel in figure 3c, which is the on-dwell time.

We have clarified that these are indeed on-dwell times. The text now reads: "Furthermore, our single-molecule read out revealed two on-state dwell times: a slow state with a dwell time of ~7 s, and a fast state with a dwell time of ~1.5 s, with the overall median dwell time largely governed by the relative frequency of these two states."

209 Concerning the time to measure the barcodes, it seems like barcode identification takes 10 minutes per colour/probe per round, so in total 10x3x2 = 60 minutes per FOV, while the actual measurement of the peptide interaction (phenotype reaction) takes 10 minutes per FOV. So, measuring the 16 protein variants separately would take 10*16 = 160 minutes, while measuring in parallel would take 60+10=70 minutes per FOV (assuming 1 FOV, which is probably not enough). So it seems the parallel setup offers a 160/70 = 2.3 times reduction in measurement time. And if we need two FOVs, this reduces to 160/(2*70) = 1.1. If this is correct, please mention this in the text. If this is incorrect, then please clarify in the text.

The reviewer's interpretation is indeed correct. Barcode acquisition requires 30 minutes and the phenotype measurement 5 minutes, resulting in a total acquisition time of 35 minutes for 16 variants measured in parallel. Measuring these variants individually would require 80 minutes of imaging time (16 × 5 minutes). Our workflow therefore indeed corresponds to an approximately 2.3-fold reduction in imaging time. We note that this comparison reflects imaging time only; experimental preparation is not taken into account. The text now reads " In addition, parallelisation reduces measurement time. In our implementation, measuring 16 variants in parallel requires 35 minutes of imaging time (30 min barcode acquisition + 5 min phenotype), compared to 80 minutes if measured individually, corresponding to an approximately 2.3-fold reduction".

211 It is not clear why the reduction in acquisition time would be higher for replicates.

This text has been removed.

219 Clarify what indices means here

We have clarified this text. See our response to major comment 1a.

248 Not entirely clear. Protein functional analysis of individual protein sequences then?

We have changed the text to: "It bridges the gap between high-throughput sequencing-based platforms and single-molecule protein characterisation, opening new directions for proteomics, protein engineering, and directed evolution."

389 Please justify the use of Alphafold while there is already a PDB structure.

While experimental structures exist for some ClpS, the particular ClpS used in this work (itself already having been a product of directed evolution) lacks an experimentally determined structure and has very little sequence homology (29%) with the most similar ClpS sequence (PDB: 9AYN, Mycolicibacterium smegmatis MC2 155). For these reasons, we have elected to instead model all variants with AlphaFold2.

Fig 1b: Suggestion: transient hybridization (instead of annealing)

We have changed this label.

Fig 2a: In the figure it looks as if the main strand is not continuous (after nicking). But it is expected that only one of the strands is cut and the other one remains completely intact.

We have corrected the schematic.

Fig 2b: Scalebar not defined

We now define the scale bar in the caption.

Fig 3: An example trace of the peptide binding to the ClpS would be helpful.

We have instead added a few example traces to Extended Data Fig. 3.

Fig 3c: Is it only 2 modes or 3 modes? For some it looks like three populations. If a molecule switches conformation within a trajectory, then taking the mean times would result in intermediate values.

We see 2 clear modes. The apparent 3rd mode could be real, but suffers from low numbers of data points. Therefore we are not confident to assign a mode.

Extended data Fig 1: How is the clustering done when the population is not present? Why are there dark purple molecules in between the blue and green populations.

We apologise for the lack of detail on how clustering is carried out. We have now added a description to the methods. Briefly, to get unbiased, yet automated clustering we employed a Gaussian mixture model (GMM). The model was first trained on a dataset, for which the ground-truth kinetic classes were known (Fig 2C), allowing robust estimation of the underlying distributions. After fitting, the resulting model parameters were held fixed and used to assign cluster identities to experimental data via maximum likelihood classification. This approach avoids bias from missing populations in individual datasets, as clustering is not performed de novo on each measurement but instead relies on a globally trained model. The presence of every population is therefore not required for robust clustering. The purple dots in Extended data Fig 1 correspond to a rare misclassification event due to overlap of the fitted Gaussian components. In our actual measurements of protein-variants such misclassifications are removed by the applied standard-deviation filter (see methods).

Reviewer #3 (Remarks to the Author):

I have read the paper 'Multiplexed single-molecule analysis of barcoded protein variants' by Mueller et al. The paper described a DNA barcoding procedure that allows for immobilized proteins to be positively identified during a single-molecule microscopy experiment, thus allowing for multiple protein variants to be characterized within the same experiment. The authors then use variants of plantomycetes ClpS previously reported in the literature as a proof of principle experiment. Their barcoding system is novel, well-characterized, and thoroughly analyzed. I would recommend this paper for publication.

In paragraph 4 of the section titled 'Main', the authors write that the experiments will demonstrate "…differences in kinetic behavior driven primarily by the relative abundance of each kinetic phenotype…". I think this statement suggests that the paper will be comparing the unique bimodal kinetics of each mutant, rather than comparing the unimodal kinetics caused by a single mutant against the bimodal kinetics of the each other variant. What the paper accomplishes is itself good, but I think the introduction would be clearer without this sentence accidentally giving readers incorrect expectations.

We interpret this comment to mean that the reviewer thinks we compare one variant showing a unimodal distribution with other variants showing bimodal distributions. However, we instead compare the distributions of mean dwell times across molecules for each variant. We find that most mutants display bimodal dwell time distributions, with a specific mutation (V72M) determining the extent of this bimodality. We have clarified this in paragraph 4. The text now reads: "Several variants displayed bimodal kinetics, with the V72M mutation determining the extent of this bimodality."

I would suggest that at the sentence "Combining the colour of the probe with the dwell time of its binding, allows the identification of hundreds of thousands of protein variants in the same assay" should be rewritten "…binding allows for…" and include a citation to 'A theoretical analysis of single molecule protein sequencing via weak binding spectra' by Rodriques, Marblestone, & Boyden.

We have changed the sentence and added the reference.

In paragraph 1 of the section 'Main', the following edit be made: "…revealing heterogeneous behaviors that are obscured in bulk measurements and transform[ing] our ability to probe the dynamics of biomolecular interactions."

We have made this edit.

This work employs a ClpS-SNAP fusion construct. Because of the small size of ClpS and the conformational changes to the N and C terminals reported in the literature upon binding, the SNAP domain could arguably affect binding. A comparison of ClpS binding with and without the snap tag by an orthogonal method such as BLI or SPR would strengthen this paper.

We thank the reviewer for this suggestion. While we agree that tags can in principle influence binding, prior work (PMID 36227977) shows comparable binding behaviour for related ClpS constructs without the SNAP tag. Moreover, our conclusions rely on relative comparisons across variants within the same construct, and are therefore not affected by any potential systematic influence of the tag.

In paragraph 2 of the section Main, the claim is made "In typical single-molecule imaging, the number of distinguishable species is constrained by the limited number of fluorophores that can be used simultaneously." I agree with this claim, but feel the paper would be strengthened if a citation more fully demonstrating this limitation could be provided.

We have added PMID 22404588.

I would suggest that in addition to citations 18 and 19 of Tullman et al., a citation should be included for 'Contributions from ClpS surface residues in modulating N-terminal peptide binding and their implications for NAAB development' by Callahan et al.

We have added the reference

The authors say in the first paragraph of the discussion section that they are working with variants of planctomycetes ClpS. This information should be clarified before the experimental results and a standard terminology ("ClpS") be established for the rest of the paper.

We have added this in the introduction and results section.

Paragraph 3 of the section Discussion brings up the possible role of the ClpS dynamic regime being altered during ClpA binding. The authors are right to cite reference 26, but should also consider citing and incorporating points from 'AAA+ protease-adaptor structures reveal altered conformations and ring specialization' by Kim et al. and 'A single amino acid substitution alters ClpS2 binding specificity' by Bergonzo et al.

We have added the following text, citing these papers to the discussion: "Structural and biochemical studies have shown that changes in ClpS conformation can alter substrate specificity[35,36]

Reviewer #4 (Remarks to the Author):

I co-reviewed this manuscript with one of the reviewers who provided the listed reports. This is part of the Nature Communications initiative to facilitate training in peer review and to provide appropriate recognition for Early Career Researchers who co-review manuscripts.

Reviewer #2 (Remarks to the Author):

The authors have clarified several points raised in the previous review. It is now clearer that multiple indices can be imaged without necessarily requiring sequential measurements for each variant. However, I have two remaining concerns that should be addressed before publication.

First, the calculation of the theoretical barcode capacity appears to be inconsistent. The authors state that they calculate the number of possible barcodes using the binomial coefficient with replacement, but the explicit formula is not shown. Using the standard formula for combinations with replacement, C(n + r - 1, r), and excluding the all-no-signal case, the expected number of usable barcodes would be C(n + r - 1, r) - 1, where n is the number of distinguishable signal states per index, including the no-binding state, and r is the number of indices. If three probes with three kinetic signatures are used, there are 9 observable signal classes plus one no-binding class, giving n = 10. This gives 9, 54, 219, and 5,004 possible barcodes for 1, 2, 3, and 6 indices, respectively. Similarly, for six probes with three kinetic signatures, there are 18 observable signal classes plus one no-binding class, giving n = 19. The corresponding numbers are therefore 18, 189, 1,329, and 134,595. These values appear to differ from the numbers currently stated in the manuscript, including 270, >20,000, and >300,000 barcodes. The authors should therefore check and correct these numbers, or explain more explicitly if a different counting scheme is intended.

In our barcode design, probe identities can be repeated within a barcode. For example, barcodes such as A7+A7 and A7+A8+A9 are valid. We have clarified this counting procedure by including a section to the methods. We now explicitly provide the formula used to calculate barcode scalability:

$$N(P,n) = \binom{P+n-1}{n} 3^n,$$

where P is the number of probes and n is the number of indices. Using this calculation, a barcode comprising two indices and three probes yields 54 possible barcodes, increasing to 270 barcodes for three indices and three probes. Expanding the system to six probes and six indices increases the barcode capacity to 336,798.

Second, the practical measurement time for large libraries remains insufficiently quantified. The authors now state that 16 variants can be measured in parallel in 35 min, compared with 80 min if measured individually, corresponding to a 2.3-fold reduction in imaging time. This is useful. However, the extrapolation to much larger libraries remains unclear. For example, if approximately 2,000 molecules can be resolved per field of view, then a library of 134,595 variants would require roughly 67 fields of view merely to observe one molecule per variant on average. This would correspond to approximately 67 hours of acquisition time at 1 hour per field of view, and substantially longer if multiple molecules per variant are needed for reliable statistics. Even 10 molecules per variant would require approximately 28 days of measurement time under these assumptions. The manuscript should therefore provide a more realistic quantitative estimate of the experimentally accessible library size, or moderate the claims about scalability accordingly.

We agree that number of potential barcodes and experimentally accessible library size are not equivalent, and that the latter is constrained by the number of single molecules that can be resolved within a given field of view. We have therefore added more text to further clarify this. The following text has been added to the discussion: “The number of variants that can be analysed per experiment can be limited by the number of single molecules that can be

resolved within the field of view, depending on the microscope configuration. Our conventional TIRF imaging conditions readily enable screening of libraries comprising hundreds to thousands of variants. Larger libraries could be accommodated through additional fields of view, albeit at the cost of increased imaging time. Alternative high-throughput single-molecule imaging implementations may further increase throughput”
